# A Prognostic Model of Head and Neck Cancer Based on Amino Acid Metabolism-Related Signature and Its Implication for Immunosuppressive Microenvironment

**DOI:** 10.3390/ijms241411753

**Published:** 2023-07-21

**Authors:** Xuran Li, Danni Li, Jiaojiao Li, Yiliang Chen, Zhenyu Cai, Fei Tan

**Affiliations:** 1 Department of ORL-HNS, Shanghai Fourth People’s Hospital, School of Medicine, Tongji University, Shanghai 201804, China; 2111599@tongji.edu.cn (X.L.); danny0524@tongji.edu.cn (D.L.);; 2 Department of Biochemistry and Molecular Biology, School of Medicine, Tongji University, Shanghai 201804, Chinadrcaizhenyu@tongji.edu.cn (Z.C.); 3 The Royal College of Surgeons in Ireland, D02 YN77 Dublin, Ireland; 4 The Royal College of Surgeons of England, London WC2A 3PE, UK

**Keywords:** amino acid metabolism, head and neck cancer, tumor microenvironment, immune checkpoint therapy, epigenetic drugs

## Abstract

Amino acid metabolism has been implicated in tumorigenesis and tumor progression. Alterations in intracellular and extracellular metabolites associated with metabolic reprogramming in cancer have profound effects on gene expression, cell differentiation, and tumor immune microenvironment. However, the prognostic significance of amino acid metabolism in head and neck cancer remains to be further investigated. In this study, we identified 98 differentially expressed genes related to amino acid metabolism in head and neck cancer in The Cancer Genome Atlas. Using batch univariate Cox regression and Lasso regression, we extracted nine amino acid metabolism-related genes. Based on that, we developed the amino acid metabolism index. The prognostic value of this index was validated in two Gene Expression Omnibus cohorts. The results show that this model can help predict tumor recurrence and prognosis. The infiltration of immune cells in the tumor microenvironment was analyzed, and it was discovered that the high index is associated with an immunosuppressive microenvironment. In addition, this study demonstrated the impact of the amino acid metabolism index on clinical indicators, survival of patients with head and neck cancer, and the prediction of treatment response to immune checkpoint inhibitors. We conducted several cell experiments and demonstrated that epigenetic drugs could affect the index and enhance tumor immunity. In conclusion, our study demonstrates that the index not only has important prognostic value in head and neck cancer patients but also facilitates patient stratification for immunotherapy.

## 1. Introduction

Head and neck cancer (HNC) is the seventh most common cancer worldwide [1]. It is often associated with pain, disfigurement, upper aerodigestive dysfunction, and even death. Patients with advanced HNC might still have a poor prognosis despite receiving traditional treatments, such as surgery, chemotherapy, and radiation [2]. Emerging oncotherapy, especially immunotherapy (e.g., pembrolizumab and nivolumab), have demonstrated prolonged survival in patients with recurrent and/or metastatic HNC [3]. However, only a small proportion of patients are sensitive to immunotherapy. Therefore, a novel prognostic model is needed to evaluate the response of individual patients to immunotherapy and predict treatment efficacy.

Growing evidence has demonstrated that amino acids act as not only metabolites but also metabolic regulators in supporting the growth of cancer cells [4]. For example, cancer cells selectively consume exogenous serine, which is converted into intracellular glycine and single-carbon units to construct nucleotides [5]. On the other hand, glutamine is involved in the tricarboxylic acid (TCA) cycle, antioxidant activity, and production of other important amino acids [6,7], which promote tumor cells to maintain rapid proliferation. In addition, amino acid metabolism reprogramming is critical for immune responses in the tumor microenvironment (TME) [8]. As one of the effectors of the immune system, T cells and their activation, differentiation, and function also heavily rely on amino acid metabolism [9].

Previous studies revealed that HNC is a severe immunosuppressive disease characterized by abnormal secretion of pro-inflammatory cytokines and immune cell dysfunction [10]. Immune cells are an important part of tumor stroma and play a crucial role in tumor progression [11,12]. Tumor cells can produce immunosuppressive metabolites through amino acid catabolism and create an immunosuppressive tumor microenvironment [13,14]. Crosstalk between cancer cells and nearby immune cells eventually leads to an environment that promotes tumor growth and metastasis [15]. For example, indoleamine 2, 3-dioxygenase 1 (IDO1) and tryptophan 2, 3-dioxygenase 2 (TDO2) can catalyze the kynurenine (KYN) metabolic pathway. KYN then leads to the generation of immunotolerant dendritic cells (DC) and regulatory T cells (Tregs), which foster a defective tumor immune microenvironment [16]. Moreover, catabolic products of the amino acid enzyme, interleukin-4-induced-1 (IL4I1), expressed in HNC stromal cells, can inhibit the immune reactivity of T cells [17]. Furthermore, the dysfunction and apoptosis of T cells caused by tryptophan consumption may be associated with primary anti-PD-1 resistance in HNC patients [18]. However, studies on the association between amino acid metabolism and TME in HNC are still limited.

Some believe that the effect of amino acid metabolism on the TME may be realized through the epigenetic mechanism. Epigenetic modifications of DNA and histones can regulate gene expression by manipulating chromatin accessibility to transcriptional machinery. For example, methionine could be the main substrate for methyl donor S-adenosine-L-methionine (SAM) biosynthesis in Th17 cells [19]. In addition, previous studies implied a close association between epigenetic agents and enhanced immune checkpoint therapy [20].

**Figure 1:**
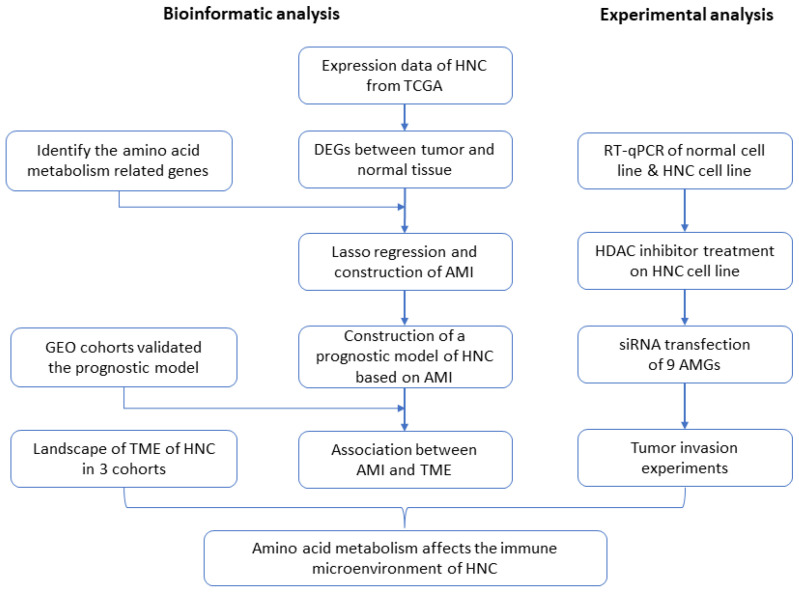
Flow chart of the experimental milestones of the current project. (HNC = head and neck cancer, AMGs = amino acid metabolism-related genes, DEGs = differentially expressed genes, TCGA = The Cancer Genome Atlas, AMI = amino acid metabolism index, GEO = Gene Expression Omnibus, TME = tumor microenvironment, HDAC = histone deacetylase).

In this study, we have established a biological index, the amino acid metabolism index (AMI), based on amino acid metabolism-related genes (AMGs). The prognostic value of this index has been systematically evaluated in The Cancer Genome Atlas (TCGA) and Gene Expression Omnibus (GEO). In addition, the potential relationship between AMI and TME, and that between AMI and immunotherapy response, have been identified and evaluated, providing a reference for personalized immunotherapy. Several in vitro experiments validated the expression of AMGs, as well as their effect on invasion. Figure 1, as the flow chart of this study, shows the design of the experiment in detail. Finally, we hold that the association between amino acid metabolism and TME in HNC may be related to the epigenetic mechanism. 

## 2. Results

### 2.1. Identification and Analysis of Differential Amino Acid Metabolism-Related Genes in Patients with Head and Neck Cancer

**Figure 2:**
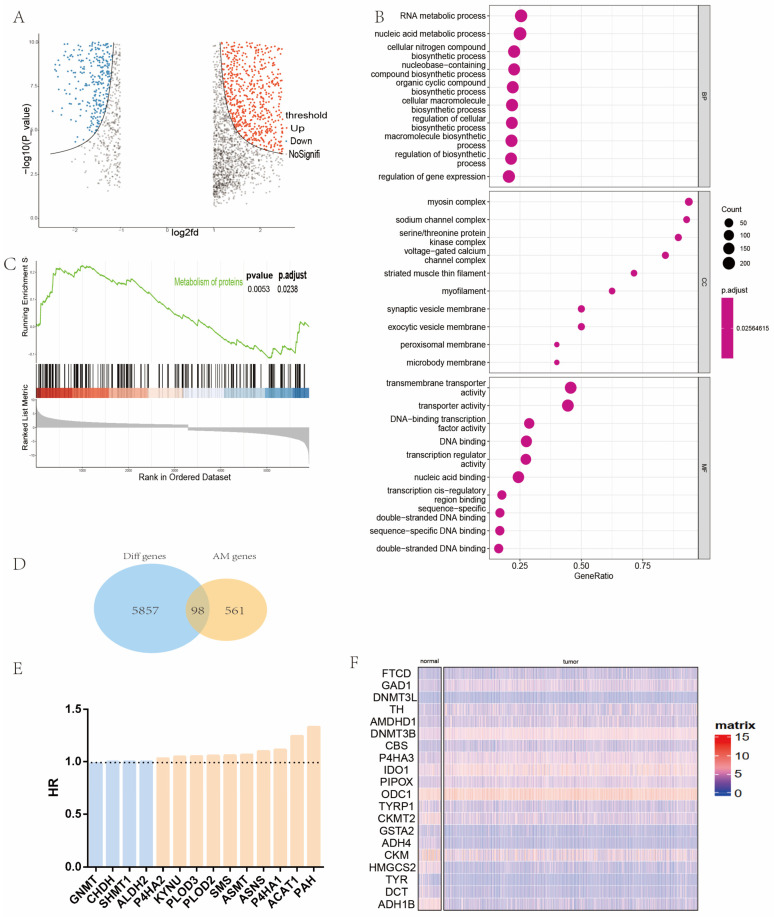
Identification of prognostic AMGs in patients with HNC. (**A**) Volcano plot of differentially expressed genes in the TCGA-HNC cohort. (**B**) GO analysis of differentially expressed genes (cellular nitrogen compound biosynthetic process, macromolecule biosynthetic process, cellular macromolecule biosynthetic process, regulation of biosynthetic process, and regulation of cellular biosynthetic process are associated with amino acid metabolism). (**C**) GSEA analysis of differentially expressed genes. (**D**) Venn diagram exhibiting 98 DEGs among AMGs. Red means positively correlated and blue means negatively correlated. (**E**) Fourteen prognostic AMGs. (**F**) Representative 20 (BP = biological process, CC = cellular component, MF = molecular function, HR = hazard ratio, HNC = head and neck cancer, AMGs = amino acid metabolism-related genes, DEGs = differentially expressed genes, GO = gene ontology, KEGG = Kyoto Encyclopedia of Genes and Genomes, TCGA = The Cancer Genome Atlas).

Firstly, |log2FC| > 1 and *p*-value < 0.05 was used as the threshold for the differential expression analysis of 531 sequencing data in the TCGA-HNC cohort. After comparing 484 head and neck cancer samples and 47 normal head and neck samples, the differentially expressed genes were collected and intersected (Figure 2A). According to GO analysis, we found that many metabolism pathways were significantly enriched in the DEGs (Figure 2B). GSEA analysis showed that protein metabolic pathways in tumor tissues were significantly enriched compared with normal tissues (Figure 2C). We obtained 98 differentially expressed metabolism-related genes (Figure 2D), and Figure 2F shows the top 20 of them. In order to identify AMGs with prognostic values, univariate Cox hazard regression analysis was applied for screening. Fourteen prognostic AMGs were screened out for further analysis (Figure 2E). We then summarized their reported mutations in HNC samples and noted that most of them have considerable mutations in patients with HNC (Figure S1). 

### 2.2. Establishment of Signature Related to Amino Acid Metabolism in Patients with Head and Neck Cancer

**Figure 3:**
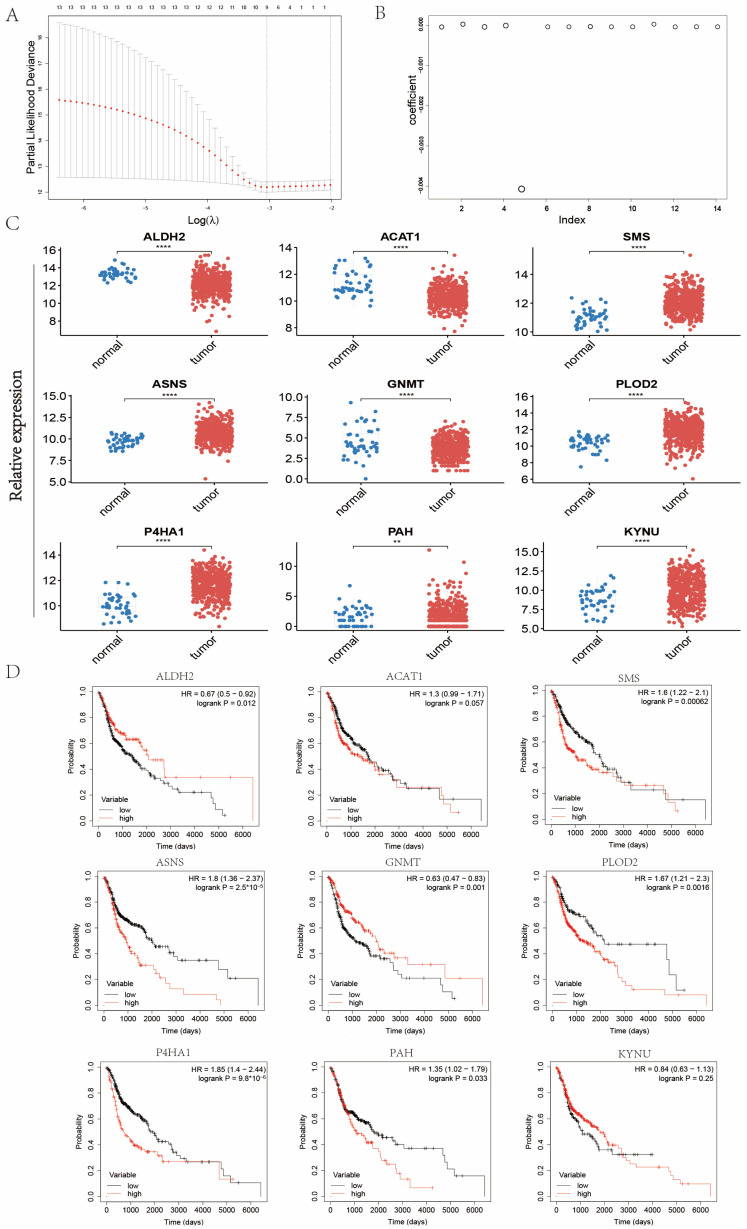
Construction of the amino acid metabolism index in the TCGA-HNC cohort. (**A**) Partial likelihood deviance for the Lasso regression. (**B**) Coefficients of Lasso regression. (**C**) Expression levels of the nine AMGs. (**D**) Kaplan–Meier (K–M) analysis based on the expression levels of the nine AMGs. (** means *p* < 0.01; **** means *p* < 0.0001, ns means no statistical significance. AMGs = amino acid metabolism-related genes.)

In order to mine the vital AMGs for establishing the metabolism feature, Lasso regression was performed to further analyze the above-discovered 14 AMGs screened by univariate Cox regression (Figure 3A,B). Ultimately, nine vital AMGs were obtained, including ALDH2, ACAT1, SMS, ASNS, GNMT, PLOD2, P4HA1, PAH, and KYNU. In addition, the expression levels of these nine AMGs and their K–M survival curves were displayed in Figure 3C,D. The expression levels of SMS, ASNS, PLOD2, P4HA1, PAH, and KYNU were upregulated in tumor samples compared with normal samples, and the remaining three genes were downregulated (Figure 3C). In Kaplan–Meier (K–M) survival analysis, the low expression group of ALDH2 and GNMT had a worse prognosis than the high expression group did, while the high expression group of SMS, ASNS, P4HA1, PLOD2, and PAH signified more impaired survival (Figure 3D). 

The correlation between the expression levels of these genes and immune cell infiltration in tumor tissues was illustrated in Supplementary Figure S2. The expression level of ALDH2 was positively correlated with B cells, CD8^+^ T cells, CD4^+^ T cells, neutrophils, and DCs. The expression level of PLOD2 was positively correlated with CD4^+^ T cells, macrophages, neutrophils, and DCs. The expression levels of GNMT, KYNU, P4HA1, and ACAT1 were also associated with the infiltration of various types of immune cells. 

**Figure 4:**
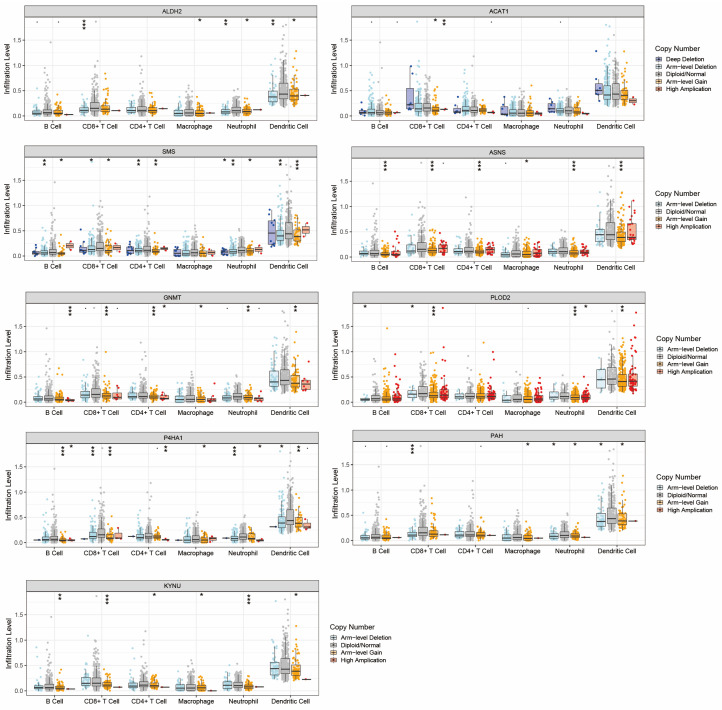
Mutations of the nine AMGs in various types of immune cells. * *p* < 0.05, ** *p* < 0.01, *** *p* < 0.001.

Mutations of these nine genes in immune cells from HNC samples were also displayed (Figure 4). KYNU, ASNS, GNMT, and P4HA1 all showed statistically significant mutations in the six dominant immune cells. SMS was mutated in five types of immune cells, while PAH, ALDH2, and PLOD2 were mutated in four types of immune cells. ACAT1 was mutated only in CD8^+^ T cells. They may largely affect the normal function of these immune cells, thereby contributing to the generation of immunosuppressive microenvironment. Therefore, our subsequent studies will focus on the tumor immune microenvironment of HNC.

Figure 4 shows mutations of nine genes in the major immune cells: B cells, T cells, macrophages, dendritic cells, and neutrophils. The main mutation types include arm-level gain, arm-level deletion, deep deletion, and high amplification (* means *p* < 0.05, ** means *p* < 0.01, *** means *p* < 0.001).

**Figure 5:**
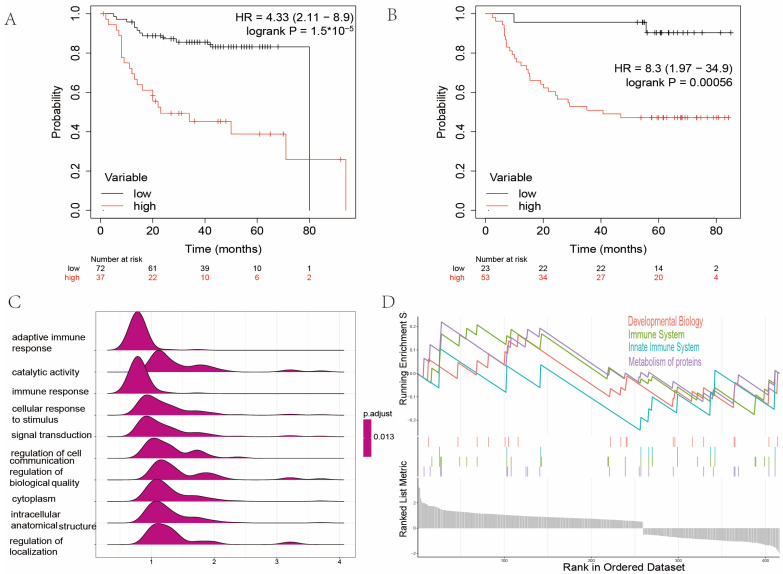
Genome enrichment analysis between high and low AMI groups. (**A**) Kaplan–Meier analysis of high and low AMI groups in GSE27020 (HR = 4.33, *p* < 0.001). (**B**) Kaplan–Meier analysis of high and low AMI groups in GSE41613 (HR = 8.3, *p* < 0.001). (**C**) GO analysis of DEGs between low and high AMI groups. (**D**) Result of GSEA between low and high AMI groups. (AMI = amino acid metabolism index, GO = gene ontology, DEGs = differentially expressed genes, GSEA = gene set enrichment analysis).

The multivariate Cox regression model was constructed with the nine AMGs, and the regression coefficients were calculated. The AMI for each HNC sample was calculated as follows: AMI = Σ Gi × βi (Gi represents the expression level of each AMG, and βi represents the corresponding regression coefficient). We adopted the TCGA cohort as a training set to develop a prognostic model. Subsequently, two GEO cohorts served as verification sets to test the predictive power of this model. The patients in GEO cohorts were divided into high and low AMI groups according to the optimal cut-off value in K–M analysis. These results showed that patients with higher AMI were more likely to suffer from tumor recurrence (HR = 4.33, *p* < 0.001, GSE27020, Figure 5A). Moreover, patients with higher AMI also had a worse prognosis (HR = 8.3, *p* < 0.001, GSE41613, Figure 5B).

### 2.3. Gene Set Enrichment Analysis between High and Low Amino Acid Metabolism Index Groups 

The GO and GSEA were performed to decipher the enriched biological processes and signaling pathways between the high and low AMI groups. The GO analysis revealed that immune-related biological processes, e.g., adaptive immune response and immune response, were enriched (Figure 5C). The GSEA demonstrated that biological functions, e.g., development biology, immune system, innate immune system, and metabolism of proteins, were enriched in the high AMI group (Figure 5D). 

### 2.4. Landscape of Immune Status and Tumor Immune Microenvironment in Head and Neck Cancer

**Figure 6:**
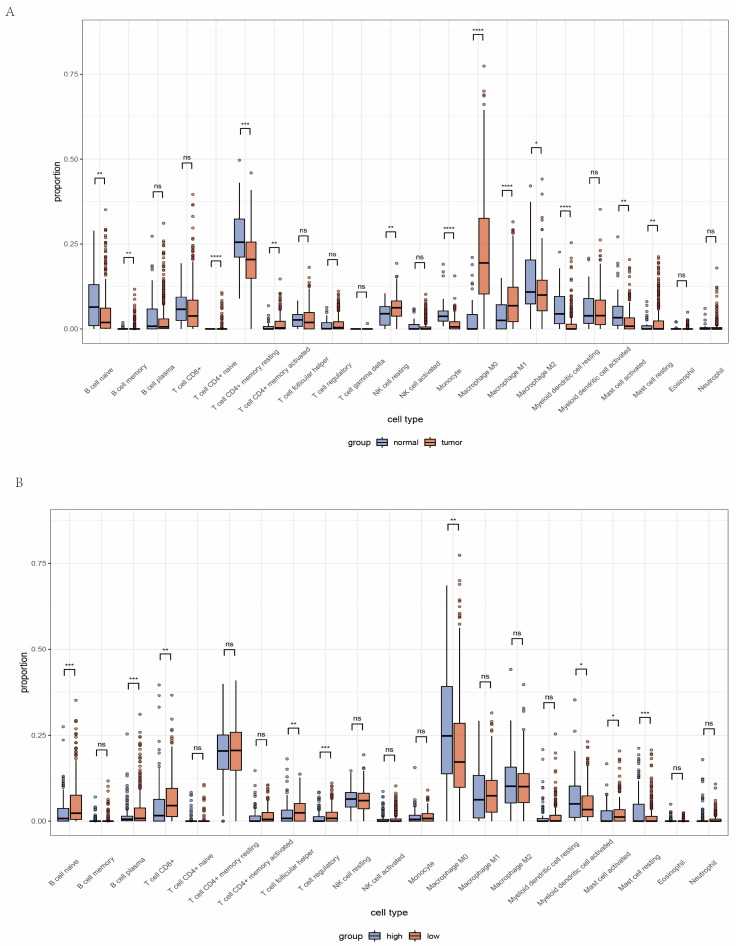
Overview of the tumor microenvironment in HNC patients. (**A**) Tumor immune microenvironment between tumor and normal tissue. (**B**) Landscape of tumor immune microenvironment between high and low AMI groups. (ns = not satistically significant, * means *p* < 0.05, ** means *p* < 0.01, *** means *p* < 0.001, **** means *p* < 0.0001, HNC = head and neck cancer).

In order to uncover the tumor microenvironment in the TCGA-HNC cohort, we performed an analysis of immune-infiltrating cells in the TME of HNC. The box plot revealed a high proportion of certain types of immune cells, i.e., resting memory CD4^+^ T cells and M0 macrophages, in the cohort (Figure S3A). Among subtypes of macrophages, immunosuppressive M2 cells infiltrated more than M1 cells. Other types of immune cells, e.g., memory B, naive CD4^+^ T cell, gamma delta T cell, and eosinophil, all showed a low infiltration proportion (Figure S3B). When comparing HNC samples with normal samples, we noticed that resting memory CD4^+^ T cell is significantly less in HNC samples, while M0 and M1 macrophages are enriched instead (Figure 6A).

### 2.5. Analysis of Immune Microenvironment between High and Low Amino Acid Metabolism Index Groups

In order to further investigate the relevance of the AMI and TME, immune cell infiltration has been assessed according to the high and low AMI groups (Figure 6B). The boxplot illustrated that an increased proportion of plasma B cells and CD8^+^ T cells was observed in the low AMI group. In addition, M0 macrophages were significantly decreased in the low AMI group. Although there was no statistical difference, immunosuppressive M2 macrophages also presented a downward trend in the low AMI group. This means that the immune response of the low AMI group is less suppressed when compared with the high AMI group, and the immune cells in the low AMI group can still play a vital anti-tumor role. Moreover, the expression of follicular helper T cells and Treg was decreased in the high AMI group. We also analyzed tumor immune cell infiltration in the GEO cohorts (Figure S4). The infiltrated immune cells in the high and low AMI groups in the GEO cohorts showed a similar trend to the TCGA cohort. In conclusion, HNC patients with high AMI may have stronger immunosuppressive TME, which might contribute to tumor progression. 

### 2.6. Potential Association between AMI and Clinical Indicators and Predictive Value of AMI for Response to Immunotherapy

**Figure 7:**
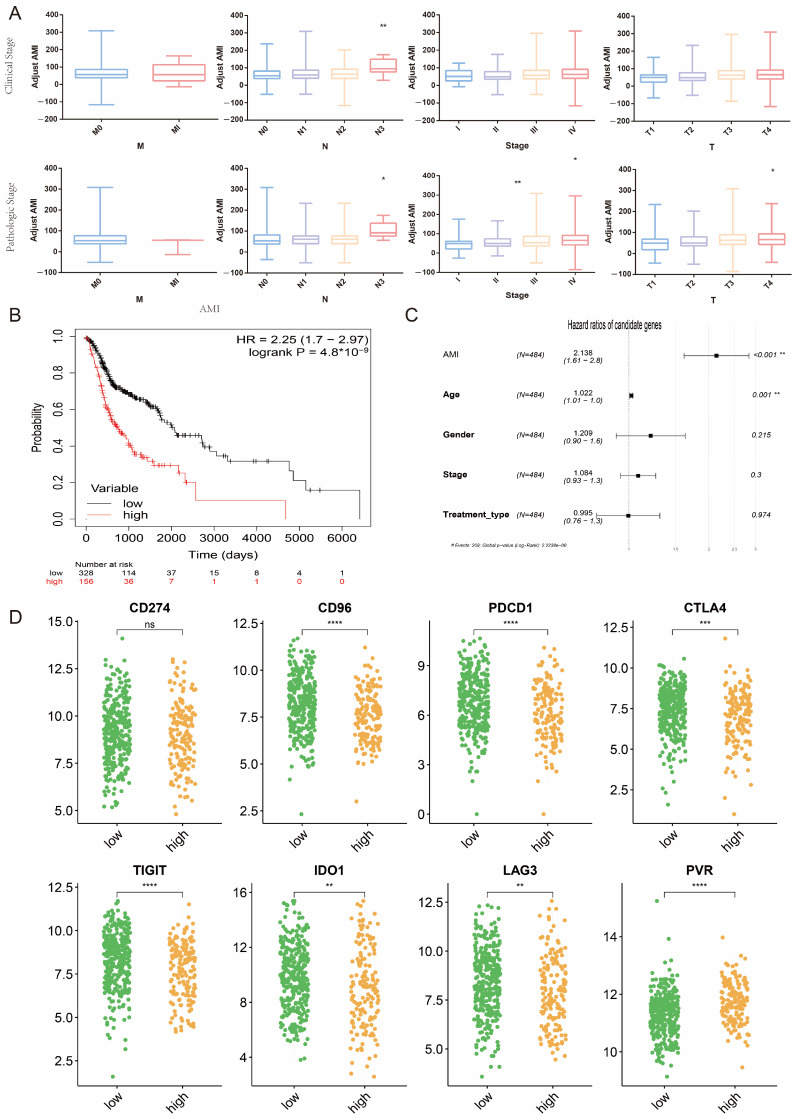
Evaluation of the association between AMI and clinical parameters and the effect of immunotherapy in HNC patients. (**A**) Comparison of AMI in different clinical and pathological grades of HNC. (**B**) K-M analysis between high and low AMI groups. (**C**) Multivariate Cox regression analysis of AMI and clinical parameters. (**D**) Expression levels of immune checkpoints in different AMI groups. (* means *p* < 0.05, ** means *p* < 0.01, *** means *p* < 0.001, **** means *p* < 0.0001, ns means no statistical significance. HNC = head and neck cancer, AMI = amino acid metabolism index.)

In order to explore the possibility of AMI in predicting clinical parameters, we investigated the relationship between AMI and clinical features using the clinical data from the TCGA cohort. In patients with HNC, both clinical staging and pathological staging showed that higher AMI is associated with more advanced tumor (T), nodal (N), and disease staging, except for metastasis (M) staging (Figure 7A).

In order to further elucidate whether AMI is associated with the survival of HNC patients, K–M analysis and multivariate Cox regression were conducted according to the high and low AMI groups. The K–M analysis showed that the high AMI group had poorer survival (*p* < 0.001) with a hazard ratio (HR) of 2.25 (Figure 7B), which was greater than the HR of any of the nine AMGs (Figure 3D). Multivariate Cox regression included AMI, age, sex, stage, and type of treatment (Figure 7C). The results suggested that only age and AMI grouping were independent prognostic predictors (*p* < 0.01). 

Although the Food and Drug Administration (FDA) has approved immunotherapy for patients with HNC, the response rate is still unsatisfactory. Depleted and dysfunctional tumor-infiltrating lymphocytes (TILs) in HNC cases are characterized by upregulation of several checkpoint markers, such as programmed cell death 1 (PD-1), lymphocyte-activating gene 3 (LAG-3), and cytotoxic T-lymphocyte-associated protein 4 (CTLA-4) [21]. Therefore, we compared the expression levels of eight immune checkpoint markers between the high and low AMI groups (Figure 7D). The results showed that the expression of PDCD1, CD96, CTLA4, TIGIT, IDO1, and LAG3 were all decreased in the high AMI group. This might render the high AMI group respond poorly to immunotherapy.

### 2.7. Potential Mechanisms of the Association between Amino Acid Metabolism Index and Tumor Immunity 

**Figure 8:**
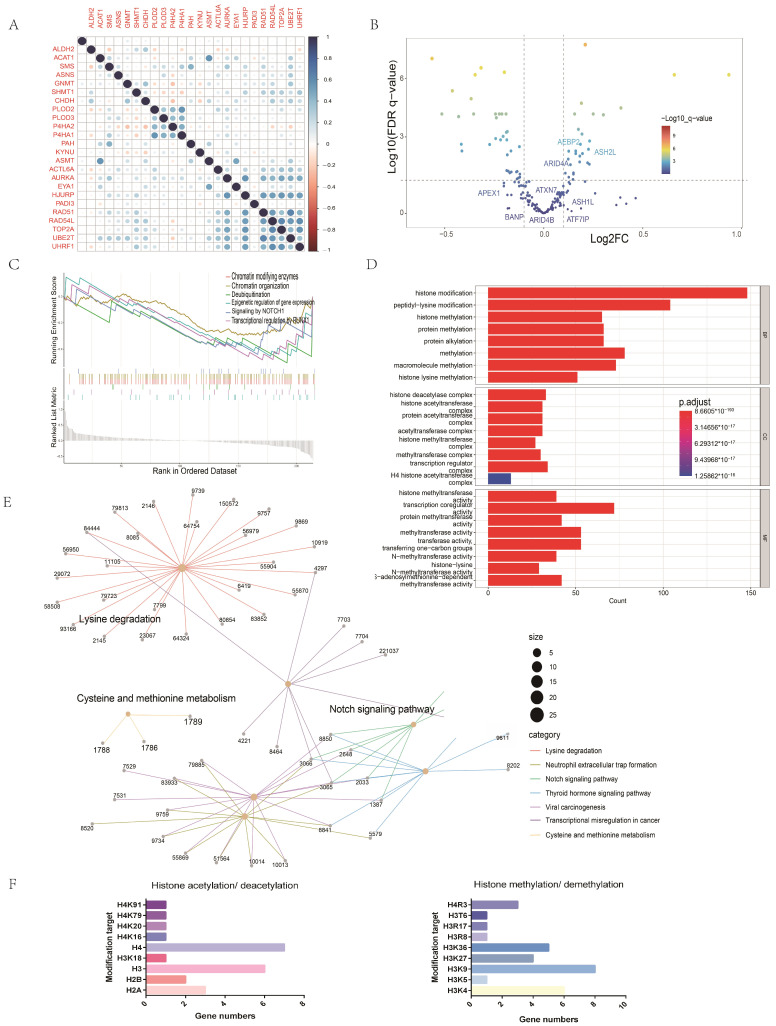
Exploration of the mechanism by which amino acid metabolism regulates tumor immunity. (**A**) Correlation analysis between AMGs and EMGs. (**B**) Volcano diagram of differentially expressed EMGs between high and low AMI groups. (**C**) GSEA of differentially expressed EMGs. (**D**) GO analysis of differentially expressed EMGs. (**E**) KEGG analysis of differentially expressed EMGs. (**F**) Major modification sites of differential EMGs. (EMGs = epigenetic-related genes, GO = gene ontology, KEGG = Kyoto Encyclopedia of Genes and Genomes, GSEA = gene set enrichment analysis.)

Previous studies have suggested that epigenetic mechanisms may be related to tumor immunosuppression status [22]. We applied univariate Cox regression to screen out epigenetic genes with prognostic values. Pearson analysis showed that there was a correlation between AMGs and prognostic epigenetic-related genes (Figure 8A). In order to investigate the underlying mechanism of the effect of amino acid metabolism on immunity, we analyzed DEGs in HNC patients according to different AMI groups. The volcano map shows epigenetically related changes in the DEGs (Figure 8B). The GO analysis indicated that these differential epigenetic genes might be associated with common epigenetic modifications, e.g., histone methylation and histone acetylation (Figure 8D). The epigenetic modification sites of these DEGs are shown in Figure 8F. KEGG analysis presented in the network plots indicated that these DEGs might be related to amino acid metabolism, e.g., lysine degradation, cysteine, and methionine metabolism (Figure 8E). Both GSEA and KEGG analyses revealed that these DEGs between high and low AMI groups were associated with the Notch signaling pathway (Figure 8C,E). Notch mutations have been proposed as predictive biomarkers for immune checkpoint blockade therapy in many cancers. In addition, we use Figure 8F to show the modification types and modification sites of these DEGs. Therefore, epigenetic mechanisms are likely to be the potential mechanism by which AMI predicts the response of immunotherapy.

### 2.8. The Expression of Nine Amino-Acid-Related Genes and the Impact on Tumor Invasion 

**Figure 9:**
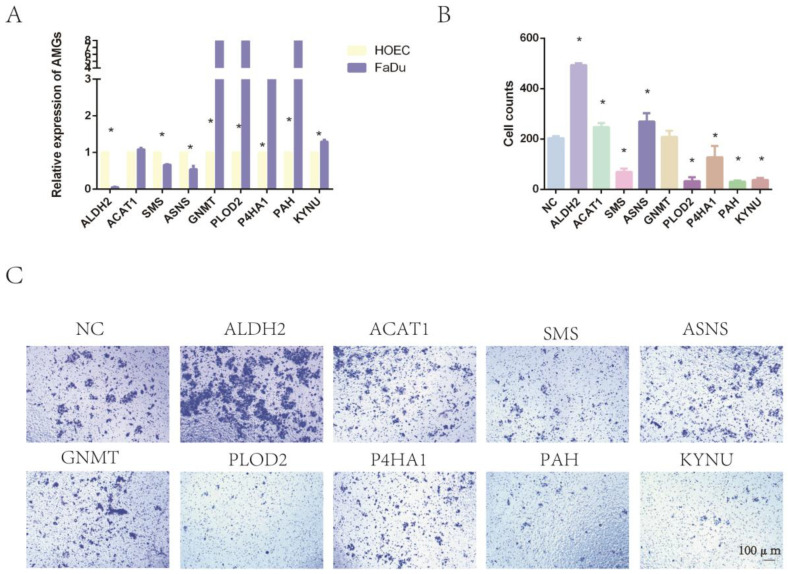
The expression of nine amino-acid-related genes and the impact on tumor invasion. (**A**) Expression of AMGs between HOEC and FaDu cells. (**B**) Cell counts of tumor invasion assay. (**C**) Impact of AMGs on tumor invasion. (* means *p* < 0.05, AMG = amino acid metabolism-related genes, HNC = head and neck cancer).

We cultured the normal oral epithelial cell line HOEC and the human pharyngeal cancer cell line FaDu. RT-qPCR was applied to compare the expression levels of AMGs between normal and tumor cells. The results showed that the relative expression levels of ALDH2, P4HA1, PAH, PLOD2, and KYNU were consistent with the results of bioinformatics analysis (Figure 9A). Furthermore, we applied siRNA to downregulate the expression level of these nine AMGs in FaDu cells. RT-qPCR was applied to confirm the reduced expression levels of these 9 genes (Figure S5).The invasion assay showed that the downregulation of ALDH2 expression significantly increased the invasion ability of tumor cells. The interference of the expression levels of SMS, P4HA1, PAH, PLOD2, and KYNU significantly decreased the invasion of tumor cells. However, the downregulation of ASNS, ACAT1, and GNMT expression had no significant impact on tumor invasion (Figure 9B,C).

### 2.9. Epigenetic Drugs Affect the Expression Level of AMGs and Tumor Immunity In Vitro

**Figure 10:**
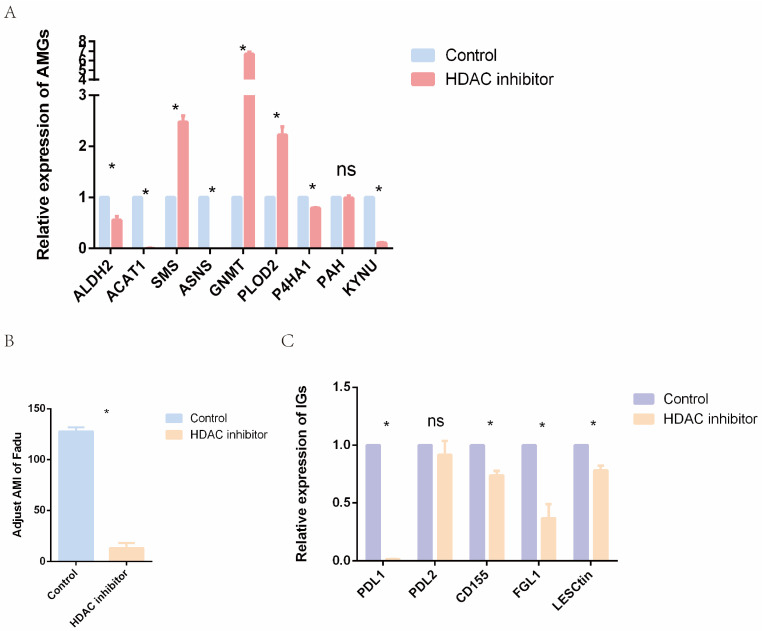
Epigenetic agents affect the relative expression levels of AMGs, immune-related genes, and AMI. (**A**) Relative expression level of AMGs in control and treatment groups. (**B**) AMI in control and treatment groups. (**C**) Relative expression level of immune-related genes in control and treatment groups. (ns = not satistically significant, * means *p* < 0.05. AMG = amino acid metabolism-related genes, AMI = amino acid metabolism index).

In order to further validate the epigenetic mechanisms underlying amino acid metabolism in HNC, RT-qPCR was applied to analyze the expression levels of AMGs. We treated human HNC FaDu cells with an effective concentration of HDAC inhibitor (SAHA, 8 μM) for 24 h. The results showed that the relative expressions of ALDH2, ACAT1, ASNS, P4HA1, and KYNU genes were significantly decreased, while the expression of SMS, GNMT, and PLOD2 genes was increased after SAHA treatment (Figure 10A). Subsequently, we further calculated adjusted AMI for the treated and control groups, and the results indicated that HDAC inhibitor significantly reduced AMI (Figure 10B). Previous analyses of this study (Figure 7B) showed that higher AMI was associated with a poorer prognosis. Therefore, HDAC inhibitors may have important therapeutic potential in HNC.

Furthermore, some immune-related genes expressed in the HNC cells were selected, and their expression levels were analyzed (Figure 10C). Apart from PDL2, all selected genes were downregulated in the HDAC inhibitor group. The downregulated PDL1 and LSECtin may contribute to relieving the dilemma of T cell dysfunction in the TME. The expression of FGL1, a ligand of LAG-3 on the surface of tumor cells, was downregulated in the SAHA group. CD155 was also significantly reduced in the treatment group. Since PDL1, CD155, FGL1, and LSECtin are all potential targets for immunotherapy, our results suggest that epigenetic drugs might influence the effect of immunotherapy.

## 3. Discussion

Metabolic reprogramming of cells is not only one of the consequences of oncogenic mutations but is also associated with tumor progression, metastasis, and recurrence [23,24,25]. Proliferating tumor cells must de novo synthesize many nitrogen-containing molecules, such as nucleotides, nonessential amino acids, and polyamines, rendering the need for nitrogen increases during tumor progression [26]. For example, glutamine consumption is greatly increased in many tumor microenvironments compared to normal tissues [27]. Furthermore, changes in metabolites can also drive changes in gene regulation. Previous studies suggested that amino acid metabolism is closely related to tumor progression also in HNC [24,28]. Therefore, we developed AMI based on the RNA expression of AMGs in the TCGA database and validated its clinical significance and predictive value in two GEO cohorts.

Using Lasso regression, we extracted nine AMGs, including ALDH2, ACAT1, SMS, ASNS, GNMT, PLOD2, P4HA1, PAH, and KYNU (Figure 3C). For example, the polymorphisms of ALDH2 are associated with HNC [29]. Downregulation of ALDH2 increases the infiltration of CD3^+^ and CD8^+^ T cells in the TME, suggesting that ALDH2 expression mediates immune system dysfunction [30]. ALDH2 and ACAT1 are involved in regulating leucine metabolism, and leucine metabolites can be utilized as substrates to enter the TCA cycle and promote macrophage activation [31]. ASNS is involved in alanine and glutamate metabolism and cell cycle control [32]. PLOD2 is associated with lysine metabolism. In addition, the expression of PLOD2 is upregulated in a variety of tumors and is associated with poor prognosis [33]. Moreover, tryptophan catabolism in macrophages suppresses the activity of the adaptive immune system, and KYNU happens to be associated with tryptophan metabolism [34].

The metabolic characteristics of tumors pose great obstacles to immune cell function. TME can reprogram the metabolism of immune cells, downregulate antigen recognition and presentation, and allow tumor cells to escape from host immune surveillance. Accumulating evidence has delineated that amino acid metabolism affects the function and number of various immune cells in the TME of HNC. In order to investigate the relevance of the AMI and TME, the infiltration of various types of immune cells has been assessed between the high and low AMI groups (Figure 6B). In HNC patients with high AMI, the proportion of CD8^+^ T cell infiltration is reduced, while that of immunosuppressive M2 macrophage is increased. One study found that more infiltration of CD8^+^ T cells in TME was associated with better clinical parameters in HNC patients, e.g., smaller tumor size and lower probability of lymph node metastasis [35]. Another study reported that higher CD8^+^ TIL levels were associated with improved overall survival (OS) and relapse-free survival (RFS) [36]. On the other hand, previous studies have indicated that metabolic reprogramming has the potential to regulate macrophage polarization, and M2 macrophages promote tumor growth by inducing immunosuppression [37]. Last but not least, the infiltration of other types of immune cells, such as follicular helper T cells and Treg, was also decreased in the high AMI group. In patients with HNC, higher levels of Treg infiltration are associated with superior OS [38]. In addition, CD8+ T suppressor in tumor-infiltrating lymphocytes may have great relevance in controlling immune system homeostasis and is associated with tumor-induced immunosuppression [39,40]. Previous studies have shown that the steroidogenic enzyme Cyp11a1 can affect the secretion function of CD8+ T lymphocyte suppressor by regulating lipid metabolism and promoting allergic reactions [41]. Therefore, amino acid metabolism is also likely to affect this particular type of immune cells, which we will pay attention to in future studies.

Recent clinical trials have demonstrated a clear survival advantage for patients with advanced HNC treated with immune checkpoint blockade [42]. Therefore, we compared the expression levels of eight immune checkpoint markers between the high and low AMI groups to see whether AMI carries predictive value for response to immunotherapy (Figure 7D). Current TME-targeted therapy mainly focuses on T cells. major examples include checkpoint blockade and chimeric antigen receptor (CAR) T-cell therapy [43]. Despite initial enthusiasm, the clinical benefits of immune checkpoint inhibitors (ICIs) vary in patients with relapsed or metastatic HNC. Positive results were experienced by 18% of patients receiving Pembrolizumab, a PD-1 inhibitor, whereas most patients with HNC were initially resistant to immunotherapy [44]. Therefore, it is important to identify those patients who are more likely to benefit from immunotherapy. Immune checkpoint signaling can create an immunosuppressive environment, which can be reversed by ICIs. Our experiments indicated that the expression levels of immune checkpoint genes, e.g., PDCD1, CD96, CTLA4, TIGIT, IDO1, and LAG3, were significantly decreased in the high AMI group (Figure 7D), suggesting that HNC patients with higher AMI may have a poorer response to immunotherapy.

Previous studies revealed a close relationship between epigenetic drugs and enhanced immune checkpoint therapy. For example, epigenetic drugs, such as HDAC inhibitors, can alter the expression of genes involved in immune checkpoints and enhance the effect of immunotherapy in HNC [45]. In addition, epigenetic agents can also improve antigen presentation by tumor cells and enhance CD8^+^ T-cell lethality [46]. Other studies implied that HDAC inhibitors, when combined with PD-1 antibodies, reduced tumor progression and improved mouse survival in a mouse melanoma model [47]. Interestingly, our in vitro cellular study also verified that an epigenetic agent, i.e., SAHA, could decrease the expression levels of immune checkpoint receptor-related genes in FaDu cells (Figure 10). Chen et al. found that FGL1 promoted LAG3-dependent T-cell suppression, and FGL1 deficiency significantly inhibited tumor growth in mouse models [48]. CD155 expressed on the surface of tumor cells can bind to TIGIT and negatively regulate NK cell function [49,50]. These results imply that epigenetic drugs can affect the expression level of AMGs, influence the effect of immunotherapy, and potentially serve as adjuvant therapeutics in HNC.

## 4. Materials and Methods

### 4.1. Transcriptome Data Preparation and Clinical Data Collection

Transcriptome expression data were obtained from the TCGA database (47 normal head and neck samples and 484 HNC samples; the sample list can be found in the Supplementary Materials). Among them, blood samples and metastases were excluded, and only tissue samples were retained. The HNC cohorts, GSE27020 and GSE41613, were downloaded from the GEO database. The clinical and pathological information of these patients was also obtained from these two databases.

### 4.2. Identification of Differentially Expressed Genes in Head and Neck Cancer

In order to identify the differentially expressed genes (DEGs) between normal head and neck samples and HNC samples, the ‘DESeq2′ R package was utilized to standardize sequencing data and screen for differential genes with the criteria set to |log2FC| > 1 and *p* adjust < 0.05. In DESeq2, *p*-values obtained from Wald tests were corrected for multiplicity using the Benjamini and Hochberg method by default.

### 4.3. Collection and Identification of Differentially Expressed Amino Acid Metabolism-Related Genes 

In order to acquire an amino acid metabolism-related gene set, three amino acid metabolism-related gene sets were downloaded from the Molecular Signature Database. The final gene set was a union of the Kyoto Encyclopedia of Genes and Genomes (KEGG) and Reactome AMGs (gene set names were listed in the Supplementary Materials). Differentially expressed AMGs in HNC were obtained using the intersection of AMGs and DEGs in R. 

### 4.4. Screening of Survival-Related Amino Acid Metabolism Genes 

The batch univariate Cox regression analysis relies on the coxph function of the ‘survival’ package in R. Thirteen survival-related AMGs were screened out by univariate Cox hazards regression analysis. 

The least absolute shrinkage and selection operator (Lasso) reduces the regression coefficient by introducing a penalty function. Lasso regression was performed on genes with *p* < 0.05 in the batch single-gene COX regression. A total of 484 patients with head and neck cancer of TCGA were applied as a training set. In this study, the Lasso regression model was conducted via ‘glmnet’ R package to select 9 optimal AMGs among all amino acid metabolism-related prognostic DEGs. The validation method is cross-validation. For each lambda value, cross-validation was performed, and the lambda value with the smallest cross-validation error was selected. In addition, the msgps function in the glmnet package was used to conduct lasso regression analysis. The Bayesian information criterion made the model simpler and less likely to lead to overfitting.

### 4.5. Construction of Amino Acid Metabolism Index in Patients with Head and Neck Cancer and Verification of Its Clinical Significance 

These optimal candidate genes were considered as variables to establish a multivariate Cox regression analysis. The variance inflation factor (VIF) and correlation coefficient of each variable in the multivariate Cox regression model were calculated to judge the possible collinearity among variables. The variables that pass the PH hypothesis and collinearity test were added to the multivariate Cox model. The multivariate Cox model calculated the regression coefficients for each variable. The AMI could be exported for each HNC patient using the following formula: AMI = Σ G_i_ × β_i_ (Gi represents the expression level of each AMG, and βi represents the corresponding regression coefficient). Two HNC cohorts from the GEO database (GSE27020 and GSE41613) were employed to validate this model.

The Kaplan–Meier (K–M) analyses were accomplished on the Kaplan–Meier Plotter website (http://kmplot.com, accessed on 21 May 2022). On the website, the optimal cutoff of AMI was obtained to divide patients with HNC into high and low AMI groups. Multiple hypothesis testing was performed using SPSS (IBM, v22), including logrank (Mantel–Cox), Breslow (Generalized Wilcoxon), and Tarone–Ware. The results of posttest *p*-values were attended in the Supplementary Materials. In order to further evaluate the feasibility of AMI in clinical settings, patients with HNC were grouped according to the optimal cutoff of AMI level, and boxplots were used to compare clinical and pathological indicators.

### 4.6. Function Enrichment Analysis between High and Low Amino Acid Metabolism Index Groups

We conducted gene set enrichment analysis (GSEA) to decode the major enriched signaling pathways and biological functions between the high and low AMI groups. The ‘ReactomePA’, ‘org.Hs.eg.db’, and ‘enrichplot’ R packages were utilized to accomplish the GESA. The top enriched signaling pathways were shown in plots.

### 4.7. Immune Landscape of Head and Neck Cancer and Significance of AMI to Tumor Immune Microenvironment and Immunotherapy 

The immune landscape of HNC was displayed via the TIMER algorithm. It serves as a comprehensive resource for the systematical analysis of immune infiltrates across diverse cancer types. The immune microenvironment analysis was performed on the TIMER 2.0 website (http://timer.cistrome.org/, accessed on 21 July 2022). This website utilizes machine learning and the CIBERSORT deconvolution algorithm to dig immune cells from a gene expression matrix in samples. Boxplots were used to show the differences in the abundance of different immune infiltrating cells in the TME of HNC samples between high and low AMI groups. In addition, the expression levels of several immune checkpoints between the high and low AMI groups were compared to predict the potential of AMI for immunotherapy efficacy.

### 4.8. Potential Mechanisms of Amino Acid Metabolism Regulating Tumor Immune Microenvironment

Based on the optimal cutoff of AMI, two groups were identified to analyze the association between amino acid metabolism and tumor immunity. In order to further explore the possible mechanisms behind this association, the limma algorithm was applied to identify DEGs between these two groups. The volcano diagram was used to demonstrate epigenetic genes that are differentially expressed between these two groups. Then, the COR function revealed the correlation between survival-related AMGS and epigenetic-related genes. We downloaded the epigenetics-related gene set on the website (https://epifactors.autosome.org/, accessed on 13 August 2022). This gene set includes 720 genes. In addition, the database provides annotations for each gene, including their modification targets and modification functions. DESeq2 package was applied to obtain the epigenetic-related genes differentially expressed between high and low AMI groups. The functions of these differentially expressed genes were then searched in the database and summarized according to the modification types. Figure 8D,F summarize the modification targets of these differential genes according to different histone modification modes.

### 4.9. Cell Culture and Agents

The FaDu cells (a hypopharyngeal squamous cell carcinoma cell line) were purchased from the Shanghai Institute of Biochemistry and Cell Biology, National Collection of Authenticated Cell Cultures, Shanghai, China. The HOEC cells (a normal human oral epithelium cell line) were purchased from Zeye Biological Co. (Shanghai, China). They were cultured in Dulbecco’s Modified Eagle’s Medium (DMEM) supplemented with 10% fetal bovine serum (FBS) and 100 U/mL penicillin/100 mg/mL streptomycin in a humidified atmosphere of 5% CO_2_ in air at 37 °C (all from Gibco, Shanghai, China). The suberoyl hydroxamic acid (SAHA), a broad-spectrum histone deacetylase (HDAC) inhibitor, was purchased from Selleck (Selleck Chemical, Houston, TX, USA). The FaDu cells were treated with 8 μM of SAHA for 24 h, while the control group was treated with 0.01% dimethyl sulphoxide (DMSO).

### 4.10. siRNA Transfection

FaDu cells were cultured in DMEM supplemented with 10% FBS and 100 U/mL penicillin/100 mg/mL streptomycin in a humidified atmosphere of 5% CO_2_ in air at 37 °C. The cells were seeded into six-well plates overnight, and the siRNA was transfected the next day using Lipofectamine 3000 according to the manufacturer’s instruction (Invitrogen, Carlsbad, CA, USA).

### 4.11. RNA Isolation and Quantitative Real-Time PCR

The total RNA of FaDu cells was extracted using Trizol reagent. The HiScript 1st Strand cDNA Synthesis Kit was used for reverse transcription PCR (RT-PCR). Quantitative real-time PCR (Q-PCR) was performed using the SYBR Green RT-qPCR Kit. All the above reagents and kits were purchased from Vazyme, Nanjing, China. The primers are listed in Supplementary Table S1. The Ct values were calculated using the ΔΔCt method, and the relative changes in mRNA levels were obtained by normalization to the glyceraldehyde phosphate dehydrogenase gene (GAPDH).

### 4.12. The Expression of Nine AMGs in Cell Lines

The FaDu and HOEC cell lines were used to further validate the expression of these 9 AMGs using quantitative real-time PCR.

### 4.13. Tumor Invasion Assay

Transwell (Millipore, Billerica, MA, USA) was used to detect the invasion ability of tumor cells. Transwell chambers were coated with 100 µL of 10% Matrigel (BD Biosciences, Franklin Lakes, NJ, USA). A total of 1 × 10^5^ cells transfected with nine different siRNAs were seeded into the upper chamber containing serum-free medium. After 24 h of incubation, the medium was discarded. Cells that had infiltrated the lower surface of the chamber were fixed with methanol for 15 min, stained with 0.1% crystal violet for 10 min, and the invaded cells were visualized using an Olympus microscope at ×200 magnification.

### 4.14. Statistical Analysis

Statistical analyses were completed using the R software (version 4.1.3, http://www.R-project.org, accessed on 4 May 2022) and GraphPad Prism (version 6). The K–M analyses were accomplished on the Kaplan–Meier Plotter website (http://kmplot.com, accessed on 21 May 2022). The univariate Cox regression analysis was conducted to screen out the survival-related AMGs. The multivariate Cox regression analysis was exploited to identify the prognostic indicators of survival. The correlation matrix was analyzed via Pearson correlation. The statistical significance was determined as *p* < 0.05.

## 5. Conclusions

We developed a prognostic model for head and neck cancer based on the expression of amino acid metabolism-related genes. Firstly, using the TCGA database, we identified the amino acid metabolism index and validated its clinical significance and predictive value in both TCGA and GEO databases. HNC patients with higher AMI scores may have worse tumor staging both clinically and pathologically. In addition, patients with higher AMI are more likely to have tumor recurrence and a worse prognosis. Secondly, after analyzing the infiltration of various immune cells, we established that high AMI is associated with the immunosuppressive microenvironment. In addition, AMI’s predictive value might also provide a reference for personalized immunotherapy. Lastly, we identified epigenetic modifications as a possible mechanism by which amino acid metabolism affects TME. Most importantly, we implemented an epigenetic drug on HNC cells and confirmed its effect on amino acid metabolism, AMI, and immune checkpoint biomarkers. Our combined bioinformatic analysis and cellular study might provide insight into the influence of amino acid metabolism on the TME of HNC while consolidating the role of epigenetic agents as adjuvant therapy for HNC patients. 

## Data Availability

The datasets used or analyzed during the current study are available from the TCGA and GEO databases.

## Supplementary Materials

The following supporting information can be downloaded at https://www.mdpi.com/article/10.3390/ijms241411753/s1.
