# Supplementary material for: A Prognostic Model of Head and Neck Cancer Based on Amino Acid Metabolism-Related Signature and Its Implication for Immunosuppressive Microenvironment
Source: Int J Mol Sci. 2023 Jul 21;24(14):11753. doi: 10.3390/ijms241411753 (PMC10380987; doi:10.3390/ijms241411753)
Supplement: Supplementary file 1 [file ijms-24-11753-s001.zip › ijms-2416918-supplementary.pdf]

Table S1. Primer sequences.

ALDH2-F	ATGGCAAGCCCTATGTCATCT
ALDH2-R	CCGTGGTACTTATCAGCCCA
ACAT1-F	TACCAGAAGTAAAGCAGCATGG
ACAT1-R	TCATTCAGTGTACTGGCATTGG
SMS-F	TAGTGGGGATGTTAATTTGGCAG
SMS-R	CCACACGTTTTTCGCATGTATTT
ASNS-F	GGAAGACAGCCCCGATTTACT
ASNS-R	AGCACGAACTGTTGTAATGTCA
GNMT-F	CTGGGGTGGACTCCATTATGC
GNMT-R	GATGACCCACTTGTCGAAGGC
PLOD2-F	CATGGACACAGGATAATGGCTG
PLOD2-R	AGGGGTTGGTTGCTCAATAAAAA
P4HA1-F	AGTACAGCGACAAAAGATCCAG
P4HA1-R	CTCCAACTCACTCCACTCAGTA
PAH-F	GCCTGCTCTGACAAACATCAT
PAH-R	TTATCTCGTGAAAGCTCATGGAC
KYNU-F	GGCTCTCCACCTAGATGAGGA
KYNU-R	GCTGCTATTTTGGCCCACTTAT
PDL2-F	ATTGCAGCTTCACCAGATAGC
PDL2-R	AAAGTTGCATTCCAGGGTCAC
CD155-F	TGGAGGTGACGCATGTGTC
CD155-R	GTTTGGACTCCGAATAGCTGG
FGL1-F	ATGGCAAAGGTGTTCAGTTTCA
FGL1-R	ACAATCTGCATACTGCCTCTTG
LSECtin-F	AGTCCTTTGGGCTGTGATTCT
LSECtin-R	AGGCGTTTGTCCTCAGCAG
PDL1-F	TGGCATTTGCTGAACGCATTT
PDL1-R	TGCAGCCAGGTCTAATTGTTTT
GAPDH-F	GGAGCGAGATCCCTCCAAAAT
GAPDH-R	GGCTGTTGCATACTTCTCATGG

Figure legend

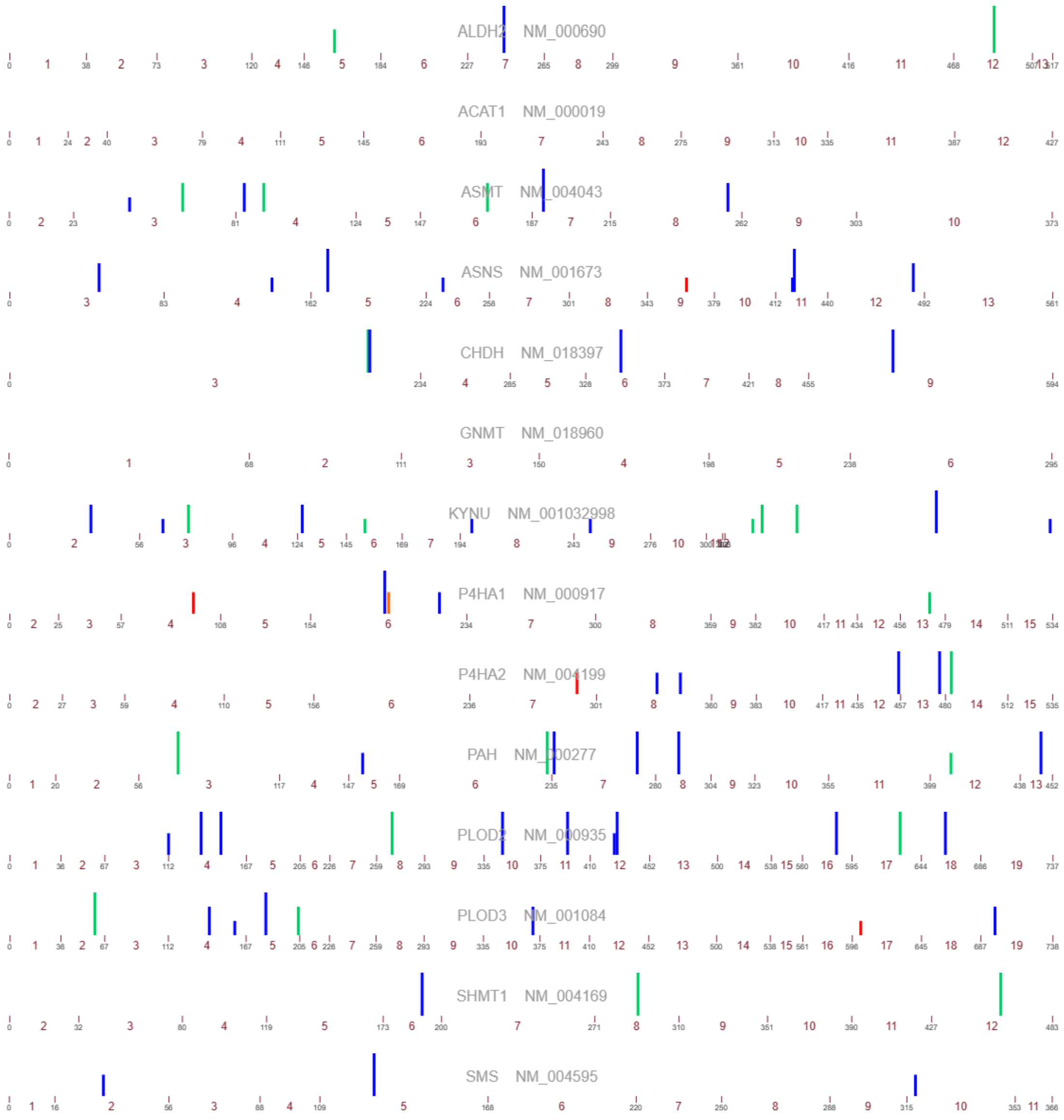

Figure S1. the mutations of AMGs in TCGA-HNC cohort. **Mutation types: .silent . missense . inframe_indel . frameshift . nonsense .**

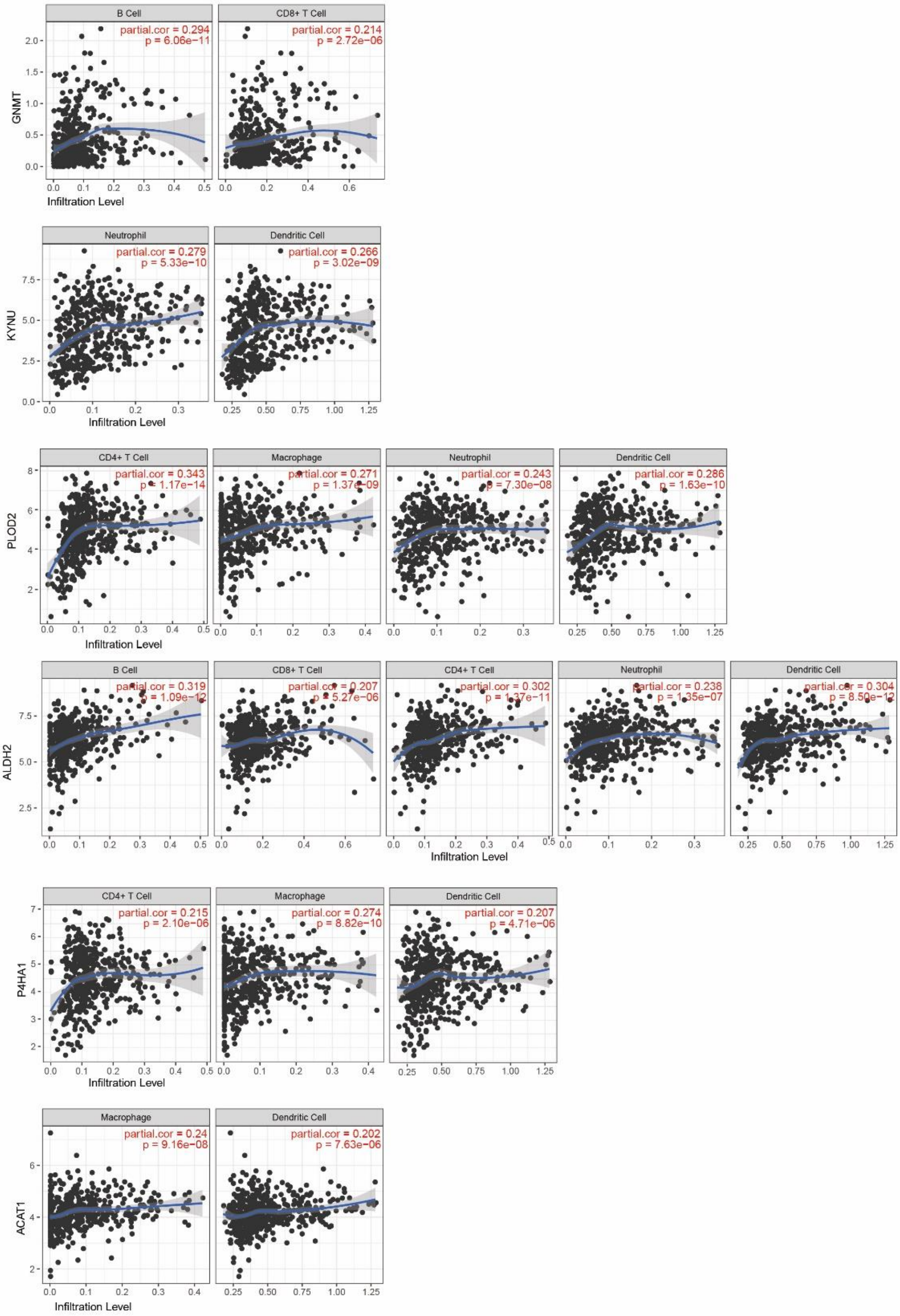

Figure S2. The correlation between these nine genes and the number of infiltration of multiple major immune cells (correlation coefficient >0.2, P value <0.05).

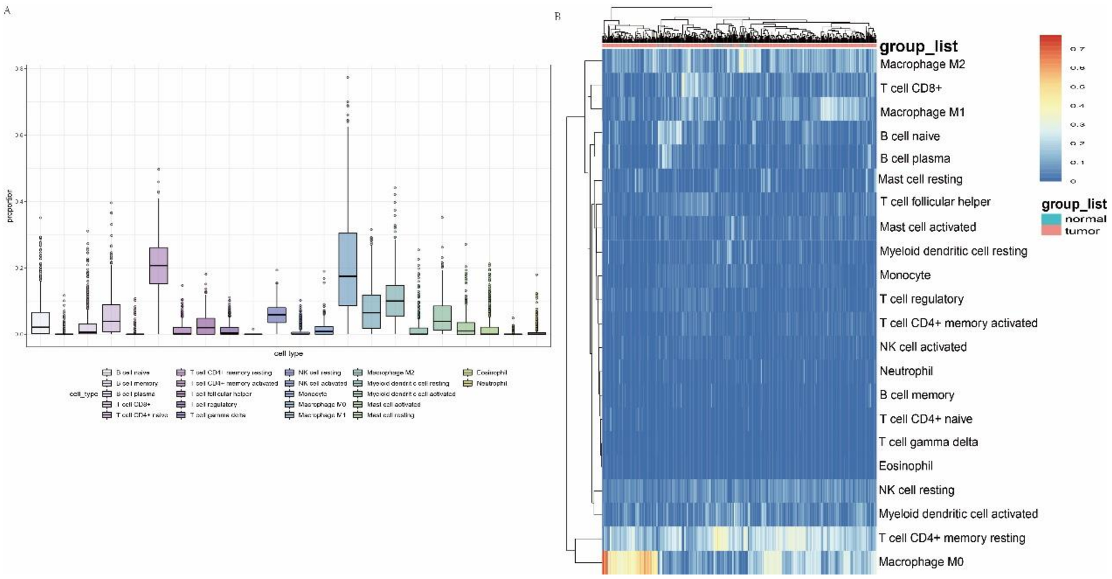

Figure S3. A, boxplot of 22 types of immune cell infiltration in HNC patients. B:heat map of immune infiltration in HNC patients.

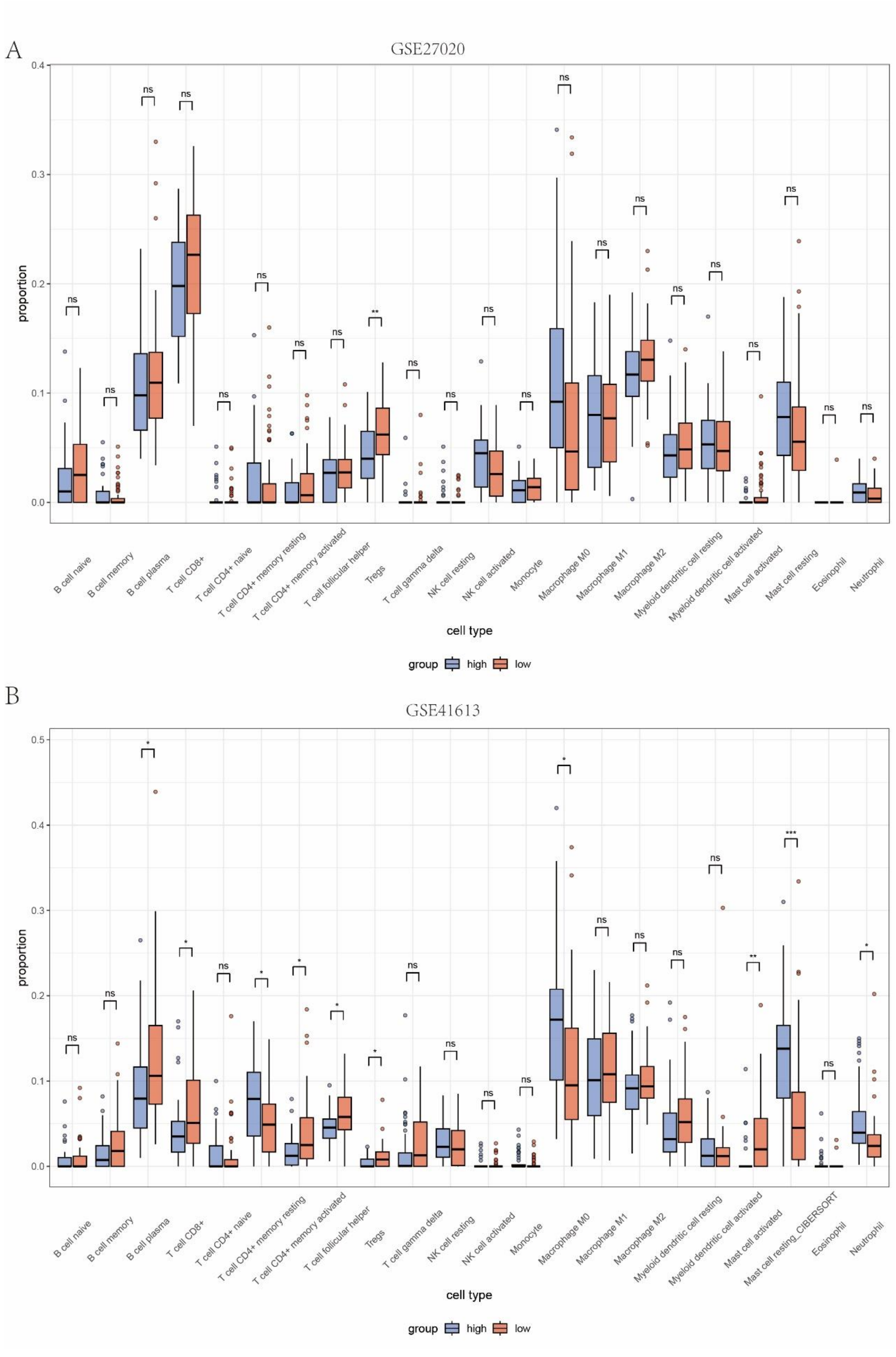

Figure S4. landscape of tumor immune microenvironment between high and low AMI groups. * $p < 0.05$, ** $p < 0.01$, *** $p < 0.001$, ns, no statistical significance.

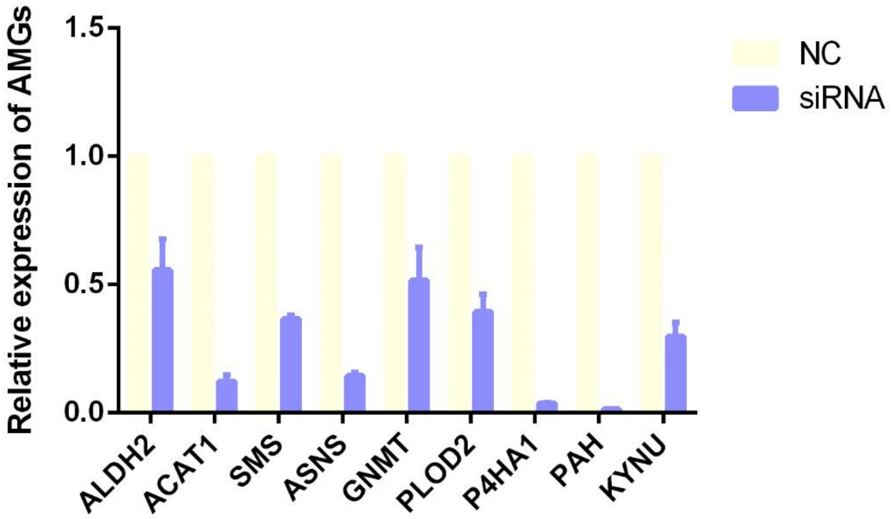

Relative expression of AMGs
1.5
1.0
0.5
0.0
NC
siRNA
ALDH2
ACAT1
SMS
ASNS
GNMT
PLOD2
P4HA1
PAH
KYNU

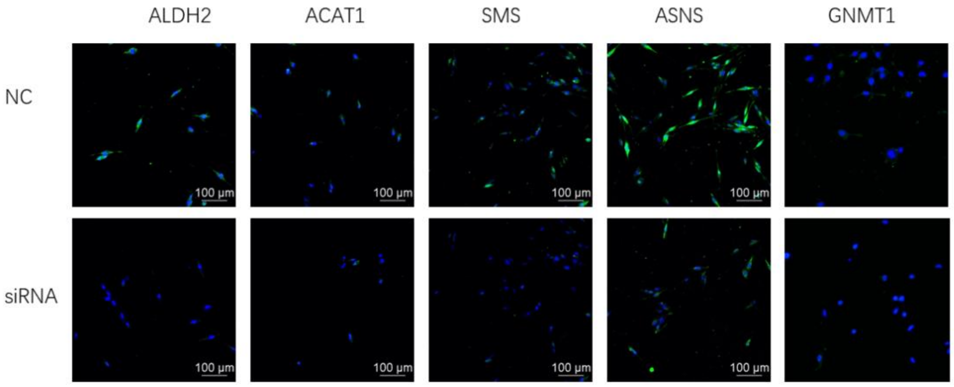

ALDH2
ACAT1
SMS
ASNS
GNMT1
NC
siRNA
100 μm

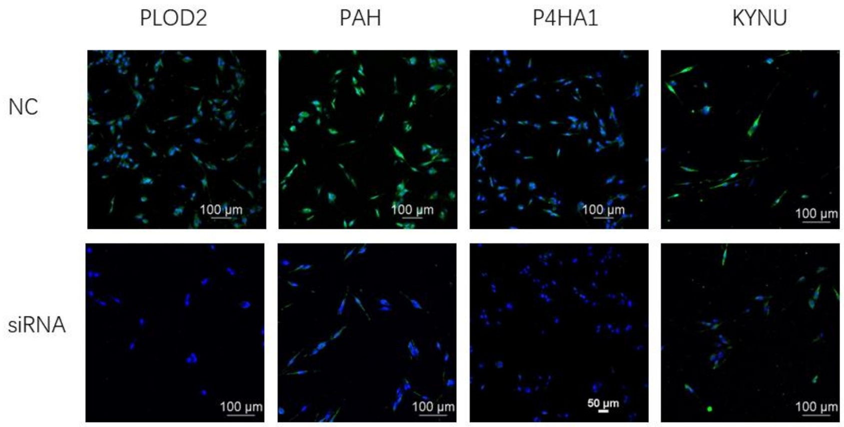

Figure S5. Verification of siRNAs suppresses AMGs expression by RT-qPCR before the invasion assay.

gene set names
KEGG_AMINOACYL_TRNA_BIOSYNTHESIS
KEGG_ALANINE_ASPARTATE_AND_GLUTAMATE_METABOLISM
KEGG_GLYCINE_SERINE_AND_THREONINE_METABOLISM
KEGG_CYSTEINE_AND_METHIONINE_METABOLISM
KEGG_VALINE_LEUCINE_AND_ISOLEUCINE_DEGRADATION
KEGG_VALINE_LEUCINE_AND_ISOLEUCINE_BIOSYNTHESIS
KEGG_LYSINE_DEGRADATION
KEGG_ARGININE_AND_PROLINE_METABOLISM
KEGG_HISTIDINE_METABOLISM
KEGG_TYROSINE_METABOLISM
KEGG_PHENYLALANINE_METABOLISM
KEGG_TRYPTOPHAN_METABOLISM
KEGG_BETA_ALANINE_METABOLISM
KEGG_TAURINE_AND_HYPOTAURINE_METABOLISM
KEGG_SELENOAMINO_ACID_METABOLISM
KEGG_GLUTATHIONE_METABOLISM
KEGG_BUTANOATE_METABOLISM

REACTOME_METABOLISM_OF_AMINO_ACIDS_AND_DERIVATIVES

Sample list
id
TCGA-CV-7103-11A-01R-2016-07

TCGA-CV-7091-11A-01R-2016-07
TCGA-CV-6960-11A-01R-2016-07
TCGA-H7-A6C5-11A-11R-A30B-07
TCGA-CV-6938-11A-01R-1915-07
TCGA-CV-7261-11A-01R-2016-07
TCGA-CV-7177-11A-01R-2016-07
TCGA-CV-7178-11A-01R-2016-07
TCGA-CV-6936-11A-01R-1915-07
TCGA-CV-7416-11A-01R-2081-07
TCGA-H7-A6C4-11A-21R-A466-07
TCGA-CV-7425-11A-01R-2081-07
TCGA-HD-A6HZ-11A-11R-A31N-07
TCGA-CV-7432-11A-01R-2132-07
TCGA-CV-6961-11A-01R-1915-07
TCGA-HD-A6I0-11A-11R-A31N-07
TCGA-CV-7245-11A-01R-2016-07
TCGA-CV-6955-11A-01R-2016-07
TCGA-CV-7101-11A-01R-2016-07
TCGA-CV-7255-11A-01R-2016-07
TCGA-CV-7423-11A-01R-2081-07
TCGA-CV-7238-11A-01R-2016-07
TCGA-CV-7440-11A-01R-2187-07
TCGA-HD-8635-11A-01R-2403-07
TCGA-CV-6935-11A-01R-1915-07
TCGA-CV-6939-11A-01R-1915-07
TCGA-CV-6962-11A-01R-1915-07
TCGA-CV-6959-11A-01R-1915-07
TCGA-CV-7250-11A-01R-2016-07
TCGA-CV-6956-11A-01R-2016-07
TCGA-CV-6934-11A-01R-1915-07
TCGA-CV-7183-11A-01R-2016-07
TCGA-CV-7424-11A-01R-2081-07
TCGA-WA-A7GZ-11A-11R-A34R-07
TCGA-CV-6933-11A-01R-1915-07
TCGA-CV-7242-11A-01R-2016-07
TCGA-CV-7235-11A-01R-2016-07
TCGA-CV-7252-11A-01R-2016-07
TCGA-CV-7097-11A-01R-2016-07
TCGA-CV-7438-11A-01R-2132-07
TCGA-CV-7406-11A-01R-2081-07
TCGA-CV-6943-11A-01R-1915-07
TCGA-CV-7434-11A-01R-2132-07
TCGA-CV-7437-11A-01R-2132-07
TCGA-CV-A45W-01A-11R-A24Z-07

TCGA-CR-7388-01A-11R-2016-07
TCGA-CR-7379-01A-11R-2016-07
TCGA-CR-7393-01A-11R-2016-07
TCGA-P3-A6T2-01A-11R-A34R-07
TCGA-CN-4737-01A-01R-1436-07
TCGA-CV-6960-01A-41R-2016-07
TCGA-T3-A92N-01A-11R-A39I-07
TCGA-CV-A6JZ-01A-11R-A31N-07
TCGA-CV-7423-01A-11R-2081-07
TCGA-CQ-6219-01A-11R-1915-07
TCGA-CV-7430-01A-11R-2132-07
TCGA-CV-A45X-01A-21R-A24Z-07
TCGA-CV-6940-01A-11R-1915-07
TCGA-CN-5361-01A-01R-1436-07
TCGA-CV-5434-01A-01R-1686-07
TCGA-BB-8601-01A-11R-2403-07
TCGA-CV-A6JD-01A-11R-A31N-07
TCGA-F7-A50I-01A-11R-A28V-07
TCGA-CN-6989-01A-11R-1915-07
TCGA-BA-A6DA-01A-31R-A31N-07
TCGA-BB-A5HZ-01A-21R-A28V-07
TCGA-CQ-A4CE-01A-11R-A266-07
TCGA-BA-A6DL-01A-21R-A30B-07
TCGA-CV-A45V-01A-21R-A24Z-07
TCGA-CV-7568-01A-11R-2232-07
TCGA-UF-A7JC-01A-21R-A34R-07
TCGA-4P-AA8J-01A-11R-A39I-07
TCGA-QK-A6IJ-01A-11R-A31N-07
TCGA-F7-A624-01A-22R-A30B-07
TCGA-HD-A633-01A-11R-A28V-07
TCGA-CV-7440-01A-11R-2132-07
TCGA-CV-A6K1-01A-11R-A31N-07
TCGA-BA-6873-01A-11R-1873-07
TCGA-CV-7410-01A-21R-2081-07
TCGA-CV-6934-01A-11R-1915-07
TCGA-CV-5971-01A-11R-1686-07
TCGA-CR-7399-01A-11R-2016-07
TCGA-BA-6872-01A-11R-1873-07
TCGA-CV-5444-01A-02R-1514-07
TCGA-UF-A7JS-01A-11R-A34R-07
TCGA-UF-A718-01A-22R-A34R-07
TCGA-CR-7394-01A-11R-2016-07
TCGA-CV-7421-01A-11R-2081-07
TCGA-CV-6003-01A-11R-1686-07

TCGA-CR-6473-01A-11R-1873-07
TCGA-UF-A719-01A-12R-A34R-07
TCGA-P3-A5QE-01A-11R-A28V-07
TCGA-CN-6022-01A-21R-1686-07
TCGA-HD-8634-01A-11R-2403-07
TCGA-CN-4725-01A-01R-1436-07
TCGA-CV-7178-01A-21R-2016-07
TCGA-CV-7428-01A-11R-2132-07
TCGA-H7-8501-01A-11R-2403-07
TCGA-HD-A6HZ-01A-12R-A31N-07
TCGA-C9-A47Z-01A-11R-A24H-07
TCGA-CV-A6K2-01A-11R-A31N-07
TCGA-CN-A642-01A-12R-A30B-07
TCGA-CV-A463-01A-11R-A266-07
TCGA-CQ-A4CB-01A-11R-A24Z-07
TCGA-CN-4739-01A-02R-1514-07
TCGA-D6-8569-01A-11R-2403-07
TCGA-CV-A464-01A-11R-A266-07
TCGA-CV-5977-01A-11R-1686-07
TCGA-CV-6441-01A-11R-1686-07
TCGA-HD-7832-01A-11R-2132-07
TCGA-F7-7848-01A-11R-2132-07
TCGA-CR-7404-01A-11R-2132-07
TCGA-CV-7238-01A-11R-2016-07
TCGA-IQ-A61I-01A-11R-A30B-07
TCGA-IQ-7630-01A-11R-2081-07
TCGA-CV-6433-01A-11R-1686-07
TCGA-TN-A7HJ-01A-12R-A34R-07
TCGA-CV-A6JY-01A-11R-A31N-07
TCGA-CV-6936-01A-11R-1915-07
TCGA-CR-7368-01A-11R-2132-07
TCGA-CQ-7071-01A-12R-A30B-07
TCGA-BA-4078-01A-01R-1436-07
TCGA-CV-7180-01A-11R-2016-07
TCGA-CN-6994-01A-11R-1915-07
TCGA-CV-A6K0-01B-21R-A31N-07
TCGA-CR-6488-01A-12R-2081-07
TCGA-CN-6997-01A-11R-2016-07
TCGA-CN-6020-01A-11R-1686-07
TCGA-CN-4729-01A-01R-1436-07
TCGA-CN-4738-01A-02R-1514-07
TCGA-CR-7374-01A-11R-2016-07
TCGA-CV-7429-01A-11R-2132-07
TCGA-CV-7183-01A-11R-2016-07

TCGA-HD-7753-01A-11R-2081-07
TCGA-BA-6870-01A-11R-1873-07
TCGA-CN-A498-01A-11R-A24H-07
TCGA-CR-7365-01A-11R-2016-07
TCGA-CN-4728-01A-01R-1436-07
TCGA-CV-7252-01A-11R-2016-07
TCGA-CQ-6220-01A-11R-1915-07
TCGA-CN-4733-01A-02R-1873-07
TCGA-CV-7255-01A-11R-2016-07
TCGA-CN-6016-01A-11R-1686-07
TCGA-BA-A4IF-01A-11R-A266-07
TCGA-P3-A6SW-01A-11R-A34R-07
TCGA-HD-7229-01A-11R-2016-07
TCGA-CQ-7063-01A-11R-2403-07
TCGA-CV-A6JE-01A-11R-A31N-07
TCGA-CQ-A4CA-01A-11R-A24Z-07
TCGA-CV-6950-01A-11R-1915-07
TCGA-CQ-6223-01A-11R-1915-07
TCGA-CV-7406-01A-11R-2081-07
TCGA-CV-7103-01A-21R-2016-07
TCGA-CV-7432-01A-11R-2132-07
TCGA-CV-5432-01A-02R-1686-07
TCGA-UF-A7JT-01A-11R-A34R-07
TCGA-HD-A6I0-01A-11R-A31N-07
TCGA-UF-A7JF-01A-11R-A34R-07
TCGA-CV-7435-01A-11R-2132-07
TCGA-CV-A45T-01A-11R-A24H-07
TCGA-CV-7413-01A-11R-2081-07
TCGA-CN-A49B-01A-31R-A24H-07
TCGA-MT-A7BN-01A-12R-A34R-07
TCGA-QK-A6II-01A-11R-A31N-07
TCGA-D6-8568-01A-11R-2403-07
TCGA-HD-7754-01A-11R-2081-07
TCGA-HD-8224-01A-11R-2403-07
TCGA-HD-A4C1-01A-11R-A24H-07
TCGA-KU-A6H7-01A-11R-A31N-07
TCGA-CN-4735-01A-01R-1436-07
TCGA-CV-5976-01A-11R-1686-07
TCGA-CV-5441-01A-01R-1514-07
TCGA-CV-7446-01A-11R-2232-07
TCGA-D6-A4Z9-01A-11R-A24Z-07
TCGA-CV-7437-01A-21R-2132-07
TCGA-F7-A623-01A-11R-A28V-07
TCGA-CR-7392-01A-11R-2016-07

TCGA-QK-A8ZB-01A-11R-A39I-07
TCGA-CN-4727-01A-01R-1436-07
TCGA-CV-5436-01A-01R-1514-07
TCGA-CV-6945-01A-11R-1915-07
TCGA-CQ-A4C7-01A-11R-A24Z-07
TCGA-BA-5559-01A-01R-1514-07
TCGA-CV-7102-01A-11R-2016-07
TCGA-CV-7104-01A-11R-2016-07
TCGA-CN-6998-01A-23R-2016-07
TCGA-MT-A51W-01A-21R-A266-07
TCGA-CN-A63V-01A-11R-A466-07
TCGA-CV-5443-01A-01R-1514-07
TCGA-D6-6517-01A-11R-1873-07
TCGA-CV-6933-01A-11R-1915-07
TCGA-CN-4723-01A-01R-1436-07
TCGA-QK-AA3J-01A-11R-A39I-07
TCGA-CR-7380-01A-11R-2016-07
TCGA-CV-7177-01A-11R-2016-07
TCGA-UP-A6WW-01A-12R-A34R-07
TCGA-QK-A6V9-01A-11R-A34R-07
TCGA-CQ-5329-01A-01R-1686-07
TCGA-F7-A61S-01A-11R-A28V-07
TCGA-CQ-6227-01A-11R-1915-07
TCGA-CV-5440-01A-01R-1514-07
TCGA-KU-A66S-01A-21R-A30B-07
TCGA-T2-A6X0-01A-11R-A34R-07
TCGA-D6-A6EN-01A-11R-A31N-07
TCGA-CR-6472-01A-11R-1873-07
TCGA-UF-A7JD-01A-11R-A34R-07
TCGA-QK-A6VB-01A-12R-A34R-07
TCGA-CQ-5330-01A-01R-1686-07
TCGA-CR-7390-01A-11R-2016-07
TCGA-CV-6941-01A-11R-1915-07
TCGA-CV-7101-01A-11R-2016-07
TCGA-CV-5973-01A-11R-1686-07
TCGA-CQ-A4CH-01A-11R-A266-07
TCGA-CQ-6228-01A-11R-1915-07
TCGA-MZ-A5BI-01A-31R-A34R-07
TCGA-F7-A620-01A-11R-A28V-07
TCGA-BA-A6DB-01A-11R-A30B-07
TCGA-CN-4730-01A-01R-1436-07
TCGA-CR-7386-01A-11R-2016-07
TCGA-P3-A5QA-01A-11R-A28V-07
TCGA-CV-6937-01A-11R-2016-07

TCGA-CN-6988-01A-11R-1915-07
TCGA-QK-A652-01A-11R-A30B-07
TCGA-CN-6012-01A-11R-1686-07
TCGA-CV-6942-01A-21R-2016-07
TCGA-CR-6467-01A-11R-1873-07
TCGA-CV-7242-01A-11R-2016-07
TCGA-CR-6470-01A-11R-1873-07
TCGA-D6-A6ES-01A-12R-A31N-07
TCGA-H7-7774-01A-21R-2081-07
TCGA-BB-4217-01A-11R-2081-07
TCGA-CN-A49A-01A-11R-A24H-07
TCGA-CV-7407-01A-11R-2081-07
TCGA-CV-A6JO-01B-11R-A34R-07
TCGA-CX-7085-01A-21R-2016-07
TCGA-F7-A61W-01A-11R-A28V-07
TCGA-CN-A6V3-01A-12R-A34R-07
TCGA-IQ-7631-01A-11R-2081-07
TCGA-T2-A6WX-01A-12R-A34R-07
TCGA-CN-5369-01A-01R-1436-07
TCGA-UF-A71E-01A-31R-A34R-07
TCGA-CV-7424-01A-11R-2081-07
TCGA-CV-6943-01A-11R-1915-07
TCGA-D6-6515-01A-21R-1873-07
TCGA-D6-6825-01A-21R-1915-07
TCGA-CN-6021-01A-11R-1686-07
TCGA-DQ-7588-01A-11R-2081-07
TCGA-F7-A50G-01A-11R-A266-07
TCGA-BA-A6DI-01A-11R-A30B-07
TCGA-CR-5250-01A-01R-1514-07
TCGA-BA-4077-01B-01R-1436-07
TCGA-CV-A45Q-01A-11R-A24H-07
TCGA-KU-A6H8-01A-21R-A34R-07
TCGA-RS-A6TP-01A-12R-A34R-07
TCGA-CV-5978-01A-11R-1686-07
TCGA-CQ-A4CG-01A-11R-A266-07
TCGA-BA-4076-01A-01R-1436-07
TCGA-MT-A67D-01A-31R-A30B-07
TCGA-D6-A6EQ-01A-11R-A31N-07
TCGA-CV-A6JT-01A-11R-A31N-07
TCGA-CV-6948-01A-11R-1915-07
TCGA-UF-A7JA-01A-12R-A34R-07
TCGA-P3-A6SX-01A-11R-A34R-07
TCGA-BB-4224-01A-01R-1436-07
TCGA-CV-A45Z-01A-21R-A24Z-07

TCGA-CV-7438-01A-21R-2132-07
TCGA-CQ-5332-01A-01R-1686-07
TCGA-BA-A4IG-01A-11R-A266-07
TCGA-UF-A7JV-01A-11R-A34R-07
TCGA-CN-6018-01A-11R-1686-07
TCGA-DQ-5625-01A-01R-1873-07
TCGA-KU-A6H7-06A-21R-A31N-07
TCGA-CN-A499-01A-11R-A24H-07
TCGA-CV-6939-01A-11R-1915-07
TCGA-CV-5970-01A-11R-1686-07
TCGA-CR-6477-01A-11R-1873-07
TCGA-H7-8502-01A-11R-2403-07
TCGA-H7-A6C4-01A-11R-A30B-07
TCGA-DQ-7591-01A-11R-2081-07
TCGA-CN-5363-01A-01R-1436-07
TCGA-CV-6955-01A-11R-2016-07
TCGA-UF-A7J9-01A-12R-A34R-07
TCGA-CR-6480-01A-11R-1873-07
TCGA-CN-5358-01A-01R-1514-07
TCGA-CQ-6218-01A-11R-1915-07
TCGA-D6-6826-01A-11R-1915-07
TCGA-F7-8489-01A-31R-2403-07
TCGA-BA-A6DE-01A-22R-A31N-07
TCGA-CR-6474-01A-11R-1873-07
TCGA-BA-5151-01A-01R-1436-07
TCGA-H7-A76A-01A-51R-A34R-07
TCGA-CV-7414-01A-11R-2081-07
TCGA-HD-8314-01A-11R-2403-07
TCGA-CV-7247-01A-11R-2016-07
TCGA-CV-6953-01A-11R-1915-07
TCGA-IQ-7632-01A-11R-2081-07
TCGA-CN-A6V7-01A-12R-A34R-07
TCGA-IQ-A61G-01A-11R-A30B-07
TCGA-F7-A622-01A-11R-A28V-07
TCGA-CV-6961-01A-21R-1915-07
TCGA-CQ-5331-01A-02R-1873-07
TCGA-D6-6823-01A-11R-1915-07
TCGA-IQ-A61J-01A-11R-A30B-07
TCGA-CV-7261-01A-11R-2016-07
TCGA-CV-7097-01A-11R-2016-07
TCGA-P3-A6T4-01A-11R-A34R-07
TCGA-CV-5435-01A-01R-1686-07
TCGA-CN-5360-01A-01R-1436-07
TCGA-CQ-5325-01A-01R-1686-07

TCGA-BB-A5HY-01A-11R-A28V-07
TCGA-CN-A6V6-01A-12R-A34R-07
TCGA-BA-A6D8-01A-31R-A31N-07
TCGA-CV-7236-01A-11R-2016-07
TCGA-CN-4742-01A-02R-1514-07
TCGA-CV-7425-01A-11R-2081-07
TCGA-CR-7395-01A-11R-2016-07
TCGA-DQ-5624-01A-01R-1873-07
TCGA-UF-A7JO-01A-11R-A34R-07
TCGA-BB-4228-01A-01R-1436-07
TCGA-CV-7089-01A-11R-2016-07
TCGA-CQ-6225-01A-11R-1915-07
TCGA-P3-A6T8-01A-11R-A34R-07
TCGA-HD-7831-01A-11R-2132-07
TCGA-CN-A63W-01A-11R-A30B-07
TCGA-UF-A71A-06A-11R-A39I-07
TCGA-CV-5431-01A-01R-1514-07
TCGA-BA-5555-01A-01R-1514-07
TCGA-CR-6491-01A-11R-1873-07
TCGA-CX-7086-01A-11R-2081-07
TCGA-F7-8298-01A-11R-2403-07
TCGA-BA-5152-01A-02R-1873-07
TCGA-CR-7364-01A-11R-2016-07
TCGA-IQ-A6SG-01A-12R-A34R-07
TCGA-CV-6951-01A-11R-1915-07
TCGA-CN-6996-01A-11R-1915-07
TCGA-CV-7100-01A-11R-2016-07
TCGA-CN-4740-01A-01R-1436-07
TCGA-CN-A63T-01A-11R-A28V-07
TCGA-CQ-5326-01A-01R-1873-07
TCGA-CQ-A4C6-01A-11R-A24Z-07
TCGA-CN-6992-01A-11R-1915-07
TCGA-CR-6481-01A-11R-1873-07
TCGA-T3-A92M-01A-31R-A39I-07
TCGA-T2-A6WZ-01A-21R-A34R-07
TCGA-P3-A6T7-01A-11R-A34R-07
TCGA-CN-5356-01A-01R-1436-07
TCGA-CV-7253-01A-11R-2016-07
TCGA-P3-A5Q6-01A-11R-A28V-07
TCGA-CN-A6UY-01A-12R-A34R-07
TCGA-CR-7370-01A-11R-2132-07
TCGA-TN-A7HI-01A-11R-A34R-07
TCGA-CQ-A4C9-01A-11R-A24Z-07
TCGA-UF-A71D-01A-12R-A34R-07

TCGA-IQ-A61H-01A-11R-A30B-07
TCGA-CR-5248-01A-01R-2016-07
TCGA-BB-4227-01A-01R-1873-07
TCGA-CN-5355-01A-01R-1436-07
TCGA-MZ-A7D7-01A-21R-A34R-07
TCGA-CV-7091-01A-11R-2016-07
TCGA-BA-5153-01A-01R-1436-07
TCGA-MT-A67A-01A-11R-A30B-07
TCGA-BB-8596-01A-11R-2403-07
TCGA-D6-A6EM-01A-21R-A31N-07
TCGA-CN-4726-01A-01R-1436-07
TCGA-CV-7099-01A-41R-2016-07
TCGA-CN-5370-01A-01R-2016-07
TCGA-CQ-7069-01A-11R-2403-07
TCGA-BA-A4IH-01A-11R-A266-07
TCGA-CR-7402-01A-11R-2016-07
TCGA-CN-5373-01A-01R-1436-07
TCGA-CN-5374-01A-01R-1436-07
TCGA-BA-A6DG-01A-21R-A30B-07
TCGA-CN-5359-01A-01R-1436-07
TCGA-CN-5365-01A-01R-1436-07
TCGA-BA-5557-01A-01R-1514-07
TCGA-CV-7416-01A-11R-2081-07
TCGA-CV-7095-01A-21R-2016-07
TCGA-BA-6868-01B-12R-1915-07
TCGA-HL-7533-01A-11R-2232-07
TCGA-CV-A45U-01A-12R-A24H-07
TCGA-HD-8635-01A-11R-2403-07
TCGA-CR-7372-01A-11R-2016-07
TCGA-CR-7373-01A-11R-2016-07
TCGA-CV-A6JU-01A-11R-A31N-07
TCGA-CQ-5334-01A-01R-1686-07
TCGA-CV-6952-01A-11R-1915-07
TCGA-CN-A63U-01A-11R-A30B-07
TCGA-CR-7371-01A-11R-2016-07
TCGA-CV-A460-01A-21R-A24Z-07
TCGA-CR-7376-01A-11R-2132-07
TCGA-CR-5249-01A-01R-1514-07
TCGA-CV-7433-01A-11R-2132-07
TCGA-CV-7235-01A-11R-2016-07
TCGA-CR-7385-01A-11R-2016-07
TCGA-CV-7418-01A-11R-2081-07
TCGA-CN-A497-01A-11R-A24H-07
TCGA-CV-7422-01A-21R-2081-07

TCGA-CV-5439-01A-01R-1686-07
TCGA-CR-6487-01A-11R-1873-07
TCGA-BA-A4II-01A-11R-A266-07
TCGA-BA-A8YP-01A-11R-A39I-07
TCGA-CV-A45Y-01A-11R-A24Z-07
TCGA-CQ-7065-01A-11R-2081-07
TCGA-DQ-5629-01A-01R-1873-07
TCGA-CQ-A4CI-01A-11R-A266-07
TCGA-QK-A8ZA-01A-11R-A39I-07
TCGA-CV-6962-01A-11R-1915-07
TCGA-DQ-5630-01A-01R-1873-07
TCGA-BB-4225-01A-01R-1436-07
TCGA-CV-5979-01A-11R-1686-07
TCGA-QK-A64Z-01A-11R-A30B-07
TCGA-WA-A7GZ-01A-11R-A34R-07
TCGA-KU-A66T-01A-11R-A30B-07
TCGA-HD-A634-01A-11R-A28V-07
TCGA-CV-6959-01A-11R-1915-07
TCGA-CR-5247-01A-01R-2016-07
TCGA-CR-6478-01A-11R-1873-07
TCGA-CN-6017-01A-11R-1686-07
TCGA-CQ-6224-01A-11R-1915-07
TCGA-QK-A6IG-01A-11R-A31N-07
TCGA-D6-6824-01A-11R-1915-07
TCGA-CV-7415-01A-11R-2081-07
TCGA-BA-5558-01A-01R-1514-07
TCGA-BA-7269-01A-11R-2016-07
TCGA-CV-5430-01A-02R-1686-07
TCGA-UF-A71A-01A-22R-A34R-07
TCGA-UF-A7JJ-01A-11R-A34R-07
TCGA-CQ-5333-01A-01R-2403-07
TCGA-CV-A461-01A-41R-A266-07
TCGA-CV-6938-01A-11R-1915-07
TCGA-CR-6482-01A-11R-1873-07
TCGA-CV-7263-01A-11R-2016-07
TCGA-CN-4731-01A-01R-1436-07
TCGA-QK-A8Z8-01A-11R-A39I-07
TCGA-TN-A7HL-01A-11R-A34R-07
TCGA-RS-A6TO-01A-32R-A34R-07
TCGA-CV-A6JN-01A-11R-A31N-07
TCGA-CQ-5327-01A-01R-1686-07
TCGA-BB-A5HU-01A-11R-A28V-07
TCGA-CV-A6JM-01A-11R-A31N-07
TCGA-CR-7383-01A-11R-2132-07

TCGA-CQ-6229-01A-11R-1915-07
TCGA-CX-7219-01A-11R-2016-07
TCGA-CN-6010-01A-11R-1686-07
TCGA-BB-4223-01A-01R-1436-07
TCGA-CR-7389-01A-11R-2016-07
TCGA-CX-7082-01A-11R-2016-07
TCGA-IQ-A61O-01A-11R-A30B-07
TCGA-BA-4075-01A-01R-1436-07
TCGA-CQ-6222-01A-11R-1915-07
TCGA-BA-5556-01A-01R-1514-07
TCGA-D6-A6EO-01A-11R-A31N-07
TCGA-CV-7248-01A-11R-2016-07
TCGA-BB-A6UO-01A-12R-A34R-07
TCGA-C9-A480-01A-12R-A24H-07
TCGA-CV-5442-01A-01R-1514-07
TCGA-CR-7382-01A-11R-2132-07
TCGA-CQ-5324-01A-01R-1686-07
TCGA-CV-7427-01A-11R-2081-07
TCGA-CQ-5323-01A-01R-1686-07
TCGA-D6-A4ZB-01A-11R-A24Z-07
TCGA-CV-A45P-01A-11R-A24H-07
TCGA-CV-5966-01A-11R-1686-07
TCGA-CN-A641-01A-11R-A30B-07
TCGA-CR-6484-01A-11R-1873-07
TCGA-CR-6492-01A-12R-2081-07
TCGA-CV-6935-01A-11R-1915-07
TCGA-CV-6436-01A-11R-1686-07
TCGA-P3-A6T0-01A-12R-A34R-07
TCGA-BA-6871-01A-11R-1873-07
TCGA-QK-A6VC-01A-23R-A34R-07
TCGA-CN-4736-01A-01R-1436-07
TCGA-P3-A5Q5-01A-11R-A28V-07
TCGA-CR-7377-01A-11R-2016-07
TCGA-P3-A6T3-01A-11R-A34R-07
TCGA-F7-A61V-01A-11R-A28V-07
TCGA-CQ-7068-01A-11R-2081-07
TCGA-CV-A465-01A-11R-A266-07
TCGA-CN-A49C-01A-11R-A24H-07
TCGA-D6-A6EP-01A-11R-A31N-07
TCGA-CV-7245-01A-11R-2016-07
TCGA-CR-6471-01A-11R-1873-07
TCGA-CR-6493-01A-11R-1873-07
TCGA-CV-6956-01A-21R-2016-07
TCGA-UF-A7JH-01A-21R-A34R-07

TCGA-CR-5243-01A-01R-1514-07
TCGA-CR-7398-01A-11R-2016-07
TCGA-CV-A45R-01A-11R-A24H-07
TCGA-IQ-A6SH-01A-12R-A34R-07
TCGA-BB-A6UM-01A-12R-A34R-07
TCGA-F7-A50J-01A-21R-A28V-07
TCGA-QK-A8Z9-01B-11R-A39I-07
TCGA-CR-7397-01A-11R-2016-07
TCGA-CR-7401-01A-11R-2016-07
TCGA-CN-5364-01A-01R-1436-07
TCGA-CV-7434-01A-11R-2132-07
TCGA-BA-4074-01A-01R-1436-07
TCGA-CN-6995-01A-31R-2016-07
TCGA-MT-A67F-01A-11R-A30B-07
TCGA-CV-7250-01A-11R-2016-07
TCGA-CQ-7072-01A-21R-A30B-07
TCGA-WA-A7H4-01A-21R-A34R-07
TCGA-CV-7090-01A-11R-2016-07
TCGA-D6-A6EK-01A-11R-A31N-07
TCGA-CN-6024-01A-11R-1686-07
TCGA-DQ-5631-01A-01R-1873-07
TCGA-QK-A6IF-01A-11R-A31N-07
TCGA-CN-6019-01A-11R-1686-07
TCGA-CR-7391-01A-11R-2016-07
TCGA-CV-7254-01A-11R-2016-07
TCGA-CV-6954-01A-11R-1915-07
TCGA-CV-7411-01A-11R-2081-07
TCGA-D6-A74Q-01A-11R-A34R-07
TCGA-CN-4722-01A-01R-1436-07
TCGA-CQ-6221-01A-11R-2081-07
TCGA-CX-A4AQ-01A-11R-A24Z-07
TCGA-BA-A6DD-01A-12R-A31N-07
TCGA-MZ-A6I9-01A-11R-A31N-07
TCGA-CR-7367-01A-11R-2016-07
TCGA-QK-A8Z7-01A-11R-A39I-07
TCGA-CN-5367-01A-01R-1436-07
TCGA-DQ-7592-01A-11R-2081-07
TCGA-CN-4734-01A-01R-1436-07
TCGA-BA-6869-01A-11R-1873-07
TCGA-IQ-A61E-01A-22R-A30B-07
TCGA-UF-A7JK-01A-11R-A34R-07
TCGA-CN-6023-01A-11R-1686-07
TCGA-CR-7369-01A-11R-2132-07
TCGA-MT-A51X-01A-11R-A266-07

TCGA-QK-AA3K-01A-11R-A39I-07
TCGA-CN-5366-01A-01R-1436-07

561 AM genes
AACS
PDHB
ALDH1B1
ACADS
PDHA1
ABAT
PDHA2
ALDH2
ACAT2
ACAT1
OXCT1
HADH
BDH1
HADHA
ACSM1
HMGCL
AKR1B10
ECHS1
ALDH9A1
GAD1
ALDH3A2
ACSM3
GAD2
ACSM4
HMGCS2
EHHADH
HMGCS1
ALDH7A1
ALDH5A1
BDH2
OXCT2
ACSM2A
ACSM5
L2HGDH
TDO2
HAAO
SRM
GGT1
GSTP1

GSTT2
GSTT1
GSTZ1
RRM2B
SMS
PGD
GSTO1
GSTA5
MGST2
LAP3
MGST1
MGST3
GSTA3
GSTM1
GPX7
GSTA4
GPX6
GSTM4
GGCT
GSTM3
GSTM2
GSTM5
GSTA1
GSTA2
GSR
GSS
RRM1
RRM2
GCLC
GSTK1
GPX5
TXNDC12
GPX1
MAOA
CPS1
P4HA3
ALDH1B1
GATM
ALDH2
OTC
PRODH2
OAT
CKM
ACY1

SAT2
CARS2
DARS2
RARS1
SARS1
VARS2
YARS2
WARS1
AARS1
FARSA
HARS1
SARS2
RARS2
NARS1
LARS2
FARSB
AARS2
YARS1
NARS2
GARS1
IARS2
KARS1
WARS2
TARS3
LARS1
VARS1
PARS2
MARS2
CARS1
IARS1
TARS2
SEPSECS
MTFMT
TARS1
DARS1
HARS2
PSTK
QARS1
EARS2
EPRS1
FARS2
MARS1
GLUD2
GFPT2

AGXT2
CPS1
GLS2
ABAT
ADSS2
ADSL
GLUD1
GLS
ASL
IL4I1
GPT
GFPT1
GPT2
DDO
ADSS1
ACY3
GAD1
GAD2
GOT2
AGXT
GLUL
NIT2
GOT1
ALDH4A1
CAD
ALDH5A1
ASS1
ASNS
PPAT
ASPA
TAT
MIF
NAA80
PNMT
DCT
LCMT1
TPO
TH
FAH
ALAS1
ALAS2
GLYCTK
MAOB
AGXT2

MAOA
AOC2
GATM
SDS
AOC3
GNMT
SHMT1
GCAT
PHGDH
DMGDH
SHMT2
SRR
PSAT1
BHMT
GLDC
DLD
GAMT
PSPH
AGXT
SARDH
AMT
CHDH
CBS
CTH
PIPOX
DAO
ADH4
ALDH3B2
ADH5
HEMK1
ADH1A
GOT2
LCMT2
TRMT11
GOT1
TYRP1
AMD1
SRM
ADI1
AHCY
DNMT1
SDS
TRDMT1
DNMT3A

DNMT3B
LDHC
IL4I1
SMS
LDHB
ENOPH1
BHMT
GOT2
CDO1
APIP
GOT1
LDHAL6B
TAT
AHCYL1
MTAP
MAT2B
LDHA
MTR
CBS
MAT1A
AHCYL2
CTH
MAT2A
MPST
LDHAL6A
DNMT3L
ADH7
DBH
ADH6
METTL2B
ADH1B
ALDH1A3
ADH1C
AOX1
ALDH1B1
ACADS
ACADSB
ABAT
ALDH2
ACADM
ACAT2
HSD17B10
ACAT1
OXCT1

ACAA2
IL4I1
HADH
MCEE
HADHB
DBT
HADHA
ALDH6A1
HMGCL
IVD
BCKDHB
ACAD8
ECHS1
ALDH9A1
ALDH3A2
PCCB
HIBCH
DLD
HMGCS2
EHHADH
HMGCS1
PCCA
ALDH7A1
HIBADH
ACAA1
OXCT2
MMUT
MCCC2
BCAT1
AUH
BCAT2
BCKDHA
MCCC1
PDHB
VARS2
IARS2
PDHA1
PDHA2
LARS1
LARS2
VARS1
BCAT1
BCAT2
IARS1

GGT7
CSAD
GGT5
GGT1
GAD1
BAAT
GAD2
GGT6
CDO1
ADO
ALDH9A1
ALDH3A2
CKMT1B
PYCR3
GAMT
PRODH
ALDH4A1
CKMT2
PYCR2
ODC1
ANPEP
GGT7
GSTO2
GGT5
G6PD
ODC1
GOT1
PRDX6
TAT
MIF
NAA80
IL4I1
DDC
SUV39H2
AASDHPPT
DLST
ALDH1B1
AASDH
ALDH2
ACAT2
PLOD2
PLOD1
TMLHE
EHMT2

ACAT1
KMT5A
HADH
SETMAR
OGDH
HADHA
BBOX1
AASS
ECHS1
ALDH9A1
ALDH3A2
KMT5B
SUV39H1
ASH1L
SETD1B
DOT1L
NSD2
EHHADH
OGDHL
SETD1A
GCDH
KMT5C
SETD7
ALDH7A1
NSD3
EHMT1
SETDB1
AADAT
SETD2
SETDB2
PLOD3
NSD1
PIPOX
SRM
AZIN2
GLUD2
GLS2
ARG1
ARG2
GLUD1
GLS
ASL
CKMT1A
P4HA2

SMS
CKB
NOS3
NOS2
NOS1
GOT2
GLUL
P4HA1
AOC1
ALDH7A1
LAP3
AGMAT
GOT1
PYCR1
ASS1
ALDH18A1
SAT1
NAGS
DAO
AMD1
MAOB
CNDP1
MAOB
MAOA
ALDH1B1
ALDH2
METTL6
ALDH3A1
BUD23
HAL
HNMT
DDC
ACY3
ALDH1A3
METTL2B
ALDH3B1
ALDH3B2
ALDH9A1
ALDH3A2
HEMK1
LCMT2
UROC1
TRMT11
AOC1

ALDH7A1
FTCD
AMDHD1
ASPA
LCMT1
HDC
ALDH3B1
MAOB
MAOA
HPD
AOX1
AOC2
TYR
HGD
METTL6
ALDH3A1
COMT
AOC3
BUD23
IL4I1
GSTZ1
DDC
ALDH1A3
ALDH3B1
ALDH3B2
MAOB
MAOA
HPD
GOT2
AOC2
ALDH3A1
AOC3
PAH
KYNU
AADAT
TPH2
ACMSD
MAOB
MAOA
IDO2
AOX1
ALDH1B1
AANAT
ALDH2

WARS1
IDO1
CAT
ACAT2
ACAT1
IL4I1
HADH
OGDH
TPH1
HADHA
DDC
AFMID
CYP1A1
CYP1A2
CYP1B1
ASMT
ECHS1
ALDH9A1
KMO
ALDH3A2
WARS2
EHHADH
OGDHL
CNDP1
SRM
ECHS1
ALDH9A1
ALDH3A2
GAD1
GAD2
AOC2
ALDH1B1
HIBCH
ABAT
EHHADH
ALDH2
ACADM
UPB1
ALDH7A1
AOC3
DPYS
DPYD
MLYCD
HADHA

SMS
GPX2
GPX3
IDH2
GPX4
IDH1
OPLAH
GCLM
GGT6
METTL6
AHCY
BUD23
SEPHS1
GGT1
SEPHS2
METTL2B
HEMK1
GGT6
LCMT2
PAPSS1
PAPSS2
TRMT11
MARS2
SCLY
AHCYL1
GGT7
GGT5
MAT2B
MAT1A
LCMT1
CBS
AHCYL2
CTH
MAT2A
MARS1
GCDH
ALDH7A1
AOC1
INMT
Multiple hypothesis testing of K-M analysis

ALDH2	Chi-square	df	significance

	Chi-square	df	significance
Log Rank (Mantel-Cox)	6.372	1	.012
Breslow (Generalized Wilcoxon)	3.745	1	.053
Tarone-Ware	4.779	1	.029

ACAT1	Chi-square	df	significance
Log Rank (Mantel-Cox)	3.609	1	.057
Breslow (Generalized Wilcoxon)	5.719	1	.017
Tarone-Ware	5.035	1	.025

SMS	Chi-square	df	significance
Log Rank (Mantel-Cox)	11.716	1	.001
Breslow (Generalized Wilcoxon)	15.689	1	.000
Tarone-Ware	15.217	1	.000

ASNS	Chi-square	df	significance
Log Rank (Mantel-Cox)	17.728	1	.000
Breslow (Generalized Wilcoxon)	8.466	1	.004
Tarone-Ware	11.635	1	.001

GNMT	Chi-square	df	significance
Log Rank (Mantel-Cox)	10.751	1	.001
Breslow (Generalized Wilcoxon)	11.821	1	.001
Tarone-Ware	11.724	1	.001

PAH	Chi-square	df	significance
Log Rank (Mantel-Cox)	4.557	1	.033
Breslow (Generalized Wilcoxon)	.617	1	.432
Tarone-Ware	1.611	1	.204

KYNU	Chi-square	df	significance
Log Rank (Mantel-Cox)	1.305	1	.253
Breslow (Generalized Wilcoxon)	1.391	1	.238
Tarone-Ware	1.647	1	.199

P4HA1	Chi-square	df	significance
Log Rank (Mantel-Cox)	19.556	1	.000
Breslow (Generalized Wilcoxon)	24.656	1	.000
Tarone-Ware	24.234	1	.000
PLOD2	Chi-square	df	significance
Log Rank (Mantel-Cox)	9.953	1	.002
Breslow (Generalized Wilcoxon)	7.103	1	.008
Tarone-Ware	8.244	1	.004
